# Intraglandular Off-the-Shelf Allogeneic Mesenchymal Stem Cell Treatment in Patients with Radiation-Induced Xerostomia: A Safety Study (MESRIX-II)

**DOI:** 10.1093/stcltm/szac011

**Published:** 2022-04-18

**Authors:** Charlotte Duch Lynggaard, Christian Grønhøj, Robin Christensen, Anne Fischer-Nielsen, Jacob Melchiors, Lena Specht, Elo Andersen, Jann Mortensen, Peter Oturai, Gry Hoffmann Barfod, Eva Kannik Haastrup, Michael Møller-Hansen, Mandana Haack-Sørensen, Annette Ekblond, Jens Kastrup, Siri Beier Jensen, Christian von Buchwald

**Affiliations:** 1 Department of Otolaryngology, Head and Neck Surgery and Audiology, Rigshospitalet, Copenhagen University Hospital, Copenhagen, Denmark; 2 Department of Clinical Medicine, University of Copenhagen, Copenhagen, Denmark; 3 Section for Biostatistics and Evidence-Based Research, The Parker Institute, Bispebjerg and Frederiksberg Hospital, Copenhagen, Denmark; 4 Research Unit of Rheumatology, Department of Clinical Research, University of Southern Denmark, Odense University Hospital, Odense, Denmark; 5 Department of Clinical Immunology, Rigshospitalet, Copenhagen University Hospital, Copenhagen, Denmark; 6 Department of Oncology, Rigshospitalet, Copenhagen University Hospital, Copenhagen, Denmark; 7 Department of Oncology, Herlev Hospital, Copenhagen University Hospital, Copenhagen, Denmark; 8 Department of Clinical Physiology, Nuclear Medicine and PET, Rigshospitalet, Copenhagen University Hospital, Copenhagen, Denmark; 9 AGiR, Department of Geoscience, Aarhus University, Aarhus C, Denmark; 10 Department of Ophthalmology, Rigshospitalet, Copenhagen University Hospital, Copenhagen, Denmark; 11 Cardiology Stem Cell Centre, The Heart Centre, Rigshospitalet, Copenhagen University Hospital, Copenhagen, Denmark; 12 Department of Dentistry and Oral Health, Aarhus University, Copenhagen, Denmark

**Keywords:** stem cells, mesenchymal stem cells, clinical trials, xerostomia, cancer

## Abstract

No effective therapy exists for the most common long-term side effect of radiation therapy for head and neck cancer (HNC)—xerostomia. The objective was to evaluate safety and provide proof of concept for efficacy of allogeneic adipose tissue-derived mesenchymal stem/stromal cells (AT-MSCs) injected into the major salivary glands of irradiated patients. This open-label, first-in-human, phase 1b, and single-center trial was conducted with repeated measurements days 0, 1, 5, and 30 and 4 months. Eligible patients with objective and subjective signs of radiation-induced salivary gland damage after treatment of oropharyngeal squamous cell carcinoma stages I-II (UICC 8) were enrolled. Twenty-five million cryopreserved AT-MSCs were injected into each submandibular and 50 million AT-MSCs into each parotid gland. Data were collected on adverse events, unstimulated and stimulated whole saliva (UWS and SWS) flow rates and saliva composition, patient-reported outcomes (EORTC QLQ-H&N35 and Xerostomia Questionnaire [XQ]), blood samples and salivary gland scintigraphy. Data were analyzed using repeated measures linear mixed models. Ten patients (7 men, 3 women, 59.5 years [range: 45-70]) were treated in 4 glands. No treatment-related serious adverse events occurred. During 4 months, UWS flow rate increased from 0.13 mL/minute at baseline to 0.18 mL/minute with a change of 0.06 (*P* = .0009) mL/minute. SWS flow rate increased from 0.66 mL/minute at baseline to 0.75 mL/minute with a change of 0.09 (*P =* .017) mL/minute. XQ summary score decreased by 22.6 units (*P* = .0004), EORTC QLQ-H&N35 dry mouth domains decreased by 26.7 (*P* = .0013), sticky saliva 23.3 (*P* = .0015), and swallowing 10.0 (*P* = .0016). Our trial suggests treatment of the major salivary glands with allogenic AT-MSCs is safe, warranting confirmation in larger trials.

## Graphical Abstract

**Figure:**
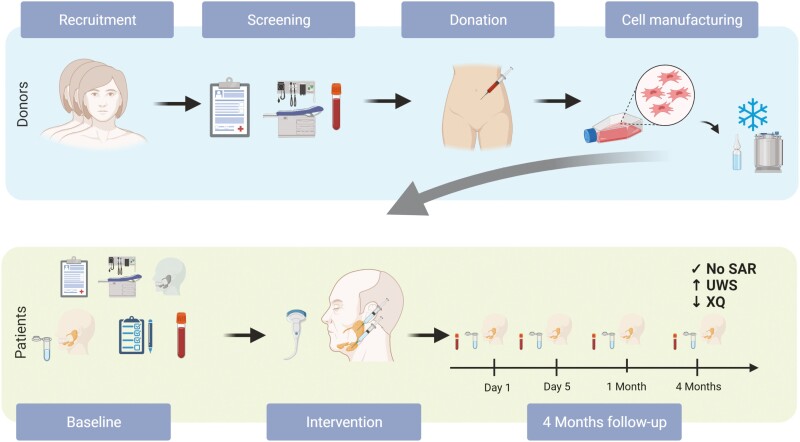

**Lessons Learned**

- Injection of an allogeneic “off-the-shelf” AT-MSC therapy into both the submandibular and parotid glands of irradiated patients is feasible.
- No treatment-related serious adverse events occurred at 4 months follow-up after treatment with AT-MSC drug in 10 patients with previous oropharyngeal squamous cell carcinoma with radiation-induced salivary gland hypofunction and xerostomia.
- A significant and clinically meaningful increase in unstimulated and stimulated whole saliva flow rate was observed along with decrease in xerostomia with improved patient-reported quality of life.
- Intraglandular treatment of the major salivary glands with allogeneic AT-MSCs is appropriate in larger randomized controlled trials.

**Significance Statement**

No efficient treatment exists to alleviate the burden of radiation-induced salivary gland hypofunction and xerostomia in patients with previous head and neck cancer. This first-in-human study evaluated the safety of and early efficacy of injecting an allogeneic “off-the-shelf” adipose tissue-derived mesenchymal stem/stromal cells (AT-MSC) product from healthy donors into both the submandibular and parotid glands of irradiated patients. We demonstrate that an allogeneic AT-MSC therapy is safe and feasible and has a tendency toward clinical efficacy in patients with radiation-induced salivary gland hypofunction and xerostomia. Our findings indicate that intraglandular treatment of the major salivary glands with allogeneic AT-MSCs is appropriate in larger randomized controlled trials testing the safety and efficacy of its applicability in the clinical setting, potentially alleviating treatment-related morbidity in patients with HNC.

## Introduction

Despite major improvements in radiation therapy for head and neck cancer (HNC), the most prevalent long-term complications are radiation-induced salivary gland hypofunction and xerostomia.^1,2^ In Western countries, the overall 5-year relative survival rate for patients with HNC is 50%-65%; thus, thousands of HNC survivors are living with treatment-related morbidity.^3,4^ The major salivary glands (SGs) are highly radiosensitive; ionizing radiation leads to reduced saliva flow rate and changes in both the composition and function of saliva.^5,6^ Consequently, patients may suffer from dental decay, oral infections, difficulties with speaking, chewing, and swallowing, sleep disturbance, worsened nutritional state, and impaired quality of life (QoL).^7,8^ Current treatments for radiation-induced salivary gland hypofunction and xerostomia are sparse and have no impact on the degeneration of the major SGs. Therapies aim with little success to stimulate the residual capacity of the SGs or to add topical oral lubrication of short duration.^9,10^ The pathophysiology behind radiation injury of SGs is multifactorial and includes an acute and late response that may continue for years, characterized by chronic inflammation, acinar, local progenitor and stem cell loss and the development of fibrosis and impaired environment for surviving acinar cells.^11-13^

Mesenchymal stem/stromal cells (MSCs) are abundant in all vascularized body tissues and are defined by their ability in vitro to adhere to plastic, to differentiate into mesodermal lineage and by having a specific set of surface markers.^14-16^ In vivo, MSCs have been suggested to primarily act by secreting a variety of cytokines and growth factors which facilitate regeneration, immunomodulation, angiogenesis, and antifibrosis and support local progenitor and stem cells.^16^ Preclinical studies indicate that MSCs can restore radiation damaged lesions, and regenerate radiation-damaged SGs to produce more saliva through the release of hepatocyte growth factor, vascular endothelial growth factor, cyclooxygenase-2, and matrix metalloproteinase-2 and by increasing the density of blood vessels in the SGs.^17-20^. In a recent randomized trial, we found that autologous adipose tissue-derived mesenchymal stem/stromal cells (AT-MSCs) injected into the submandibular glands of patients with radiation-induced xerostomia to be safe with a promising tendency to restore submandibular gland function.^21^ Allogeneic cells have advantages over autologous as they originate from young, healthy donors.

We hypothesized that intraglandular allogeneic AT-MSC treatment of the submandibular and parotid glands in patients with radiation-induced damage of the salivary glands after oropharyngeal squamous cell carcinoma (OPSCC) treatment is safe and will show preliminary signs of efficacy through increased salivary flow rates.

## Methods

### Study Design and Ethics

**Figure 1.**
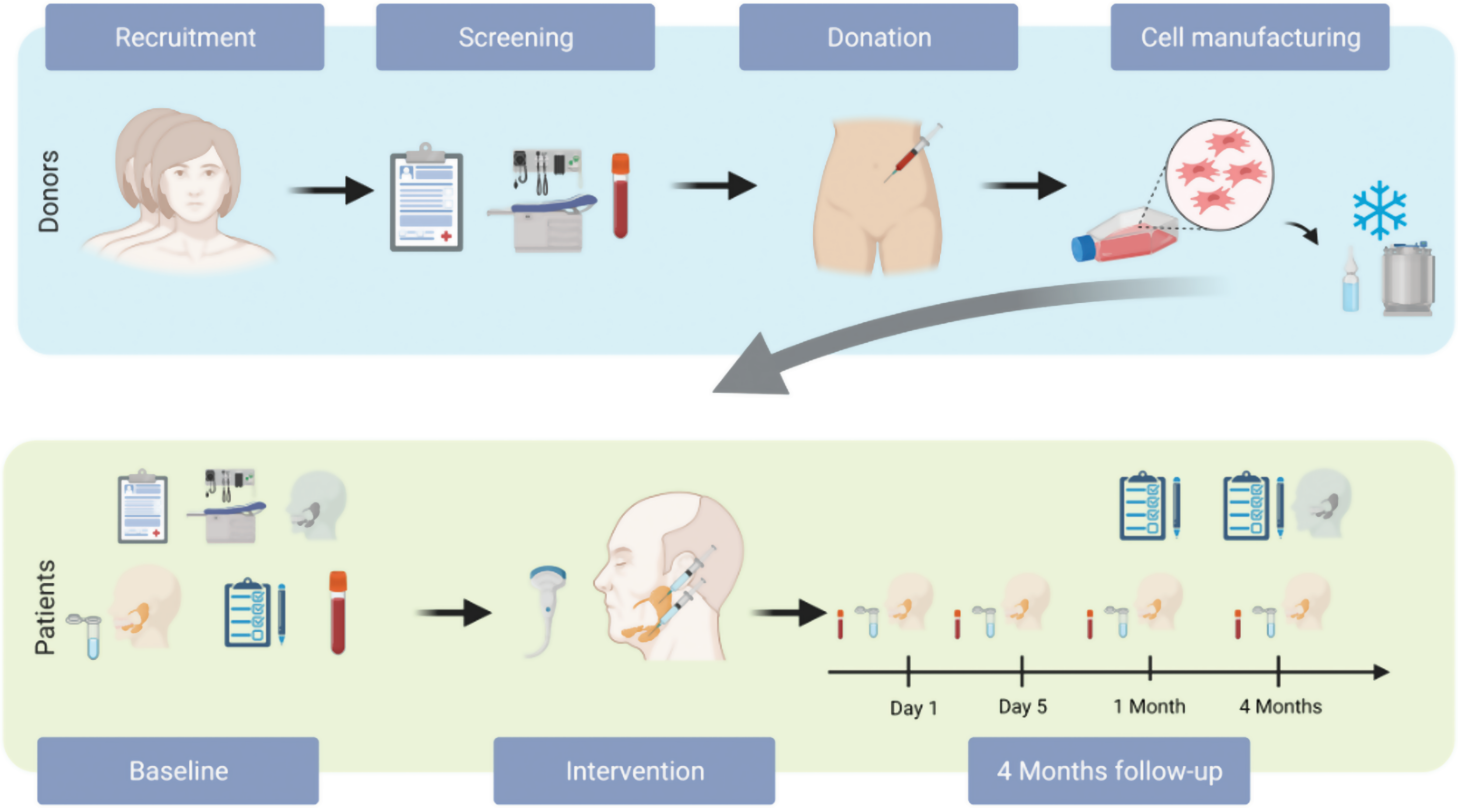
Schematic depiction of the study. Created using Biorender.com (PL22Y4W85V).

The primary objective of this investigator-initiated, first-in-human, non-randomized, single-center, open-label, phase I clinical trial was to investigate the safety of intraglandular injections of AT-MSCs into the submandibular and parotid glands of patients with radiation-induced salivary gland hypofunction and xerostomia with a 4 months follow-up (Fig. 1). Secondary objectives were to evaluate the preliminary efficacy assessed by changes in whole saliva flow rates, whole saliva composition, and patient-reported QoL. The sample size was not based on statistical power calculations, but 10 was deemed adequate for a phase I trial.

The trial was conducted according to the original protocol and complied with the Declaration of Helsinki and was approval obtained from the National Committee on Health Research Ethics (H-1808924) and the Danish Medicines Agency (Eudra-CT 2018-003856-19). The trial protocol was registered at ClinicalTrials.gov (number NCT03874572. The trial was monitored by the Good Clinical Practice Unit of Copenhagen.

### Study Participants

Patients were recruited from the Department of Otolaryngology, Head and Neck Surgery and Audiology at Rigshospitalet, the Departments of Oncology at Rigshospitalet and Herlev-Gentofte Hospital, or through self-referral triggered by media awareness. The patients had previously been treated with photon therapy (volumetric arc therapy technique) to a total dose to the tumor and lymph node metastases of 66-68 Gy given in 2 Gy per fraction with 6 fractions per week with concurrent cisplatin therapy. This treatment consistently delivers practically the full prescribed dose to the ipsilateral submandibular gland and some dose to the lower portion of the ipsilateral parotid gland. Main inclusion criteria comprised patients of both sexes between 18 and 75 years, with previous OPSCC stages I-II (The Union for International Cancer Control 8th, UICC 8), with symptoms and objective measures of radiation damage of the SGs with a minimum of 2 years without relapse after radiation therapy. Main exclusion criteria were cancer in the previous 4 years (not including OPSCC and basal cell carcinomas), xerogenic medications, penicillin or streptomycin allergy, other diseases of the SGs, any previous stem cell therapy or SG surgery, alcohol abuse, smoking, or pregnancy (A full list of eligibility criteria is provided in Supplementary Material, eMethods 1). Patients were screened for human immunodeficiency virus (HIV), syphilis, hepatitis B and C, and kidney and liver disease.

We recruited 10 healthy control participants to compare the flow rates and composition of whole saliva The control participants fulfilled same criteria as the patients and were matched for age (±5 years), sex, race, and education. All participants provided written informed consent.

### Study Treatment and Assessments

All patients received without any anesthesia ultrasound-guided injection of an AT-MSC suspension of 50 million AT-MSCs/mL with 25 million AT-MSCs in each submandibular and 50 million in each parotid gland using a 23 G × 2 2/5 KDM disposable hypodermic needle performed by the same 2 surgeons (C.D.L. and J.M.). AT-MSCs were deposited in 2 areas of the parotid glands to secure equal distribution of the suspension. The study dose was based on a previous trial, injecting 2.8 million autologous AT-MSCs/cm^3^ into the submandibular glands, yielding a total of 50-100 million AT-MSCs with no serious adverse event (SAE) and adjusted for inclusion of the parotid gland.^21^ The slightly increased AT-MSCs dose/cm^3^ was both with the attempt to increase the effect and due to the use of allogeneic AT-MSCs. Consequently, and especially due to inclusion of the parotid gland, the total administered dose was increased as above described. The intervention containing patient-unmatched allogeneic AT-MSCs was manufactured by Cardiology Stem Cell Centre from lipoaspirate obtained from 3 healthy female donors (22-26 years old), according to Good Manufacturing Practice as previously described.^22,23^ Each study batch contained cells from 1 donor and the patients only received cells from 1 donor each. The AT-MSCs drug was injected within 10 minutes after thawing in a 37 °C water bath in the outpatient clinic without any further processing. Lipoaspirates were obtained from healthy donors signing an informed consent in compliance with the Declaration of Helsinki. Donor eligibility was determined by donor interviews and testing for infectious disease markers HIV, hepatitis B and C, syphilis, and human T-cell lymphotropic virus (HTLV I/II). AT-MSCs were expanded in automated closed bioreactor systems (Quantum Cell Expansion System, Terumo BCT) with human platelet lysate as growth supplement (Sexton Biotechnologies). AT-MSCs were cryopreserved, 50 million/mL in CryoStor CS10 (10% dimethyl sulfoxide [DMSO])) or CS5 (5% DMSO) (BiolifeSolutions) and stored in nitrogen dry-storage until clinical use. Release criteria were viral safety (donor serology/NAT), sterility (including test for contamination of bacteria, fungus, mycoplasmas and endotoxins), cell number and viability (>80%), and immunophenotypical characterization of cells by flow cytometry (stable positive markers CD90, CD105, CD73 >80% and negative markers <3% CD45, <5% human leukocyte antigen (HLA)-DR). Ten-month stability of release criteria and cell function after storage were documented. All donors were HLA-A, B, -C, -DRB1, -DRB3/4/5, -DQA1, -DQB1, -DPA1, and -DPB1 typed by qPCR. Each treatment unit was based on 1 donor only. As this was the first time for a cryopreserved and allogeneic AT-MSC treatment of major SGs in humans, the trial was designed to start with excipient CS5 for the first 5 patients and CS10 for the last 5 patients (Supplementary Material, Fig. S1).

Patient follow-up occurred on day 1 after the intervention and at day 5, 1 month, and 4 months. The primary endpoint was treatment-related SAEs after 4 months evaluated by Common Terminology Criteria for Adverse Events, CTCAE vs 5.0 ( Supplementary Material, Figs. S1 and S2). To be open for all potential SAEs, specific events were not stated in the protocol. The secondary endpoints comprised change in salivary flow rates, whole saliva composition, quality of life (the European Organization for Research and Treatment of Cancer Quality of Life Questionnaire Head and Neck Module (EORTC QLQ-H&N35) and Xerostomia Questionnaire) and salivary gland function measured by scintigraphy (full description in Supplementary Material, eMethods 2).^24,25^ Measurements of saliva flow rates and saliva collection were performed using sialometry (drooling method) at every visit.^26,27^ Participants were asked to drink a minimum of 2 L of water the day before a visit. Sialometry was performed between 10:00 a.m. and 12:00 p.m. by the same doctor in the same room. At the 4 months follow-up visit, the performance of sialometry by the same doctor was monitored by a project nurse, who was blinded to the baseline values for extra validity of the results. Participants were to refrain from drinking, eating, smoking, and oral hygiene for a least 1 hour before sialometry. After 5 minutes of rest, participants swallowed 1 mouthful (15-20 mL) of refrigerator-cold water to cleanse the mouth, and unstimulated whole saliva (UWS) was collected for 10 minutes in a pre-weighed plastic cup. Chewing of a tasteless paraffin wax pellet (Ivoclar Vivadent) was used to collect stimulated whole saliva (SWS). After 1 minute of initial chewing the patients swallowed, and the saliva produced the following 5 minutes was collected with the patient chewing and spitting out the freshly produced saliva at their own pace. The saliva samples were snap frozen in liquid nitrogen and stored at −80 °C within 15 minutes from collection and stored pending analyses. Saliva flow rate (mL/minute) was estimated by dividing the saliva sample volume (1 g of saliva equals 1 mL) by the collection time (minutes). UWS and SWS samples from patients and healthy controls were analyzed for sodium, chloride, potassium, and phosphate concentrations.^28,29^ Inorganic anions chloride (Cl^−^) and phosphate (PO_4_^3−^) concentrations were analyzed using chromatography. Inorganic cations sodium (Na^+^) and potassium (K^+^) concentrations were analyzed using Inductively Coupled Plasma Mass Spectrometry (full description in Supplementary Material, eMethods3). Screening for HLA antibodies was performed on Luminex 100 System. Screening for HLA-antibodies (IgG) was detected using the LABScreen Mixed (One Lambda) and if positive, subsequent specification was performed using LABScreen Single Antigen (One Lambda). Mean fluorescence intensity (MFI) >1000 was defined as positive.

### Statistical Analysis

The objectives of the repeated measurements designs were to make inferences about the expected values of the observations, namely, the average change from baseline in our sample. As described in the prespecified Statistical Analysis Plan (found in Supplementary Material), data were analyzed from December 2020 to March 2021 using SAS version 9.4 software (SAS Studio), with the particular outcome variable (*Y*i) as a dependent variable, using a (multilevel) repeated measures mixed effects model with participants as a random effects factor, and time (days; 5 levels) as a fixed effect factor based on a restricted maximum likelihood model. This statistical model holds all between-time comparisons for all assessment points up to 4 months from baseline/day 0 (including baseline) and allows for evaluation of the average change, as well as the trajectory over time from baseline to 4 months follow-up. For the purpose of sensitivity, we also analyzed the patients with missing data “as observed” comparing differences between baseline and four months by Wilcoxon ranks test presented as medians with interquartile ranges.

## Results

Of 17 patients screened, 10 eligible patients (3 women, 7 men, aged 45 to 70 years) received ultrasound-guided injection in 4 major SGs from April 29, 2019 to January 8, 2020 (Table 1). All patients were followed for 4 months, but due the SARS-CoV-2 pandemic the last 2 patients were seen after 3 months rather than 4 months after approval of the protocol amendment by the National Committee on Health Research Ethics and the Danish Medicines Agency. No protocol deviations or violations occurred during the 4 months trial period.

**Table 1.** Baseline characteristics of patients and healthy control participants.

Variable	Patients								Healthy control participants							
	(*n* = 10)	Mean	SD	Min	Q1	Median	Q3	Max	(*n* = 10)	Mean	SD	Min	Q1	Median	Q3	Max
Age, years		61.1	7.5	45	56	64	67	70		59.5	8.8	40	54	59.5	67	71
Sex, males, no. (%)	7(70)								7(70)							
Race, Caucasian, no. (%)	10(100)								10(100)							
DSA	1(10)															
Education levels, no. (%)																
Unskilled worker	0								0							
Semi-skilled	0								0							
Skilled worker	6(60)								5(50)							
Bachelor	1(10)								1(10)							
Master	3(30)								4(40)							
DMSO high, no. (%)	5(50)															
Treatment over 2 days, no. (%)	4(40)															
Whole saliva flow rates mL/minute																
Unstimulated whole saliva flow rate		0.13	0.06	0.1	0.1	0.1	0.2	0.2		0.451	0.20	0.2	0.3	0.4	0.5	0.8
Stimulated whole saliva flow rate		0.66	0.34	0.1	0.4	0.7	0.9	1.1		2.439	1.06	0.9	1.7	2.5	3.2	4.0
XQ summary score		53.5	23.2	12.5	39.4	62.5	68.8	81.3		3.125	5.63	0.0	0.3	1.3	2.5	18.8
EORTC QLQ-H&N35 (scores 0-100)																
HNDR		73.3	30.6	12.5	66.7	66.7	100	100								
HNSS		46.7	32.2	0.0	33.3	50.0	66.7	100								
HNSW		26.7	20.7	0.0	12.5	25.0	33.3	75								
Scintigraphic uptake score (scores 0-4)																
All glands		1.7	0.8	0.5	1.1	1.8	2.0	3								
PG		2.3	0.8	1.5	2.0	2.0	2.8	4								
SMG		0.9	0.7	0.0	1.3	1.3	1.5	1.5								
Scintigraphic excretion fraction, %																
All glands		53.3	17.0	20.3	42.0	57.7	64.8	77.7								
PG		59.3	21.8	17.8	42.0	67.6	77.1	82.0								
SMG		40.3	12.1	22.8	37.0	40.4	40.9	63.1								
Saliva composition, mmol/L																
UWS chloride		21.7	8.8	13.4	17.3	18.4	25.1	42.9		17.0	2.9	13.7	14.9	16.9	17.9	24.1
SWS chloride		26.5	11.3	11.8	17.4	25.1	34.0	45.2		17.2	3.1	11.8	16.1	17.1	19.0	22.6
UWS phosphate		4.7	1.4	2.2	3.9	4.5	5.3	7.2		5.3	1.6	3.5	4.0	4.7	6.1	8.5
SWS phosphate		3.8	0.5	2.8	3.6	3.9	4.2	4.5		3.8	0.5	3.2	3.3	3.9	4.2	4.4
UWS potassium		19.1	5.0	9.7	16.6	18.3	21.9	27.2		18.4	4.2	14.6	16.2	17.6	18.4	29.6
SWS potassium		19.4	3.9	13.4	16.7	18.8	22.4	26.1		17.1	2.1	14.8	15.5	16.8	18.7	20.5
UWS sodium		8.4	5.2	2.2	5.3	7.3	9.6	18.0		6.0	2.8	2.8	4.4	5.1	7.4	11.9
SWS sodium		14.7	9.9	5.5	7.0	11.2	18.1	34.0		15.0	8.7	3.4	6.1	17.2	21.9	25.4
Primary tumor location, no. (%)																
Tonsil	10(100)															
Base of tongue	0(0)															
p16+, no. (%)	10(100)															
Cancer stage (UICC 8), no. (%)																
1	7(70)															
2	3(30)															
IMRT + concurrent cisplatin, no. (%)	10(100)															
Radiation dose SMG																
Mean Gy right SMG ± SD		55.5	12.9	34.8	46.4	59.9	66.1	67.6								
Max Gy right SMG ± SD		62.0	10.6	40.2	59.1	67.7	68.5	69.6								
Mean Gy left SMG ± SD		56.0	16.8	17.2	56.5	62.5	65.7	66.9								
Max Gy left SMG ± SD		58.1	17.5	25.0	51.9	68.7	69.1	69.4								
Radiation dose PG																
Mean Gy right PG ± SD		28.7	12.3	10.8	21.4	30.3	32.8	49.2								
Max Gy right PG ± SD		56.8	18.4	23.5	55.0	65.3	69.3	70.8								
Mean Gy left PG ± SD		25.6	14.0	5.4	15.1	26.4	31.7	49.9								
Max Gy left PG ± SD		49.6	20.6	19.3	29.2	55.9	67.3	68.7								
Smoking status, no. (%)																
Never smoker		7(70)							8(80)							
Previous 0-10 PY		1(10)							1(10)							
Previous > 10 PY		2(20)							1(10)							
Years from radiation therapy		5.5	2.2	2.0	4.3	5.5	6.8	9.0								

No patients experienced treatment-related SAEs. One patient with a history of multiple vasovagal syncopes developed a vasovagal syncope during injection of the last gland, the left parotid gland (Table 2). After recovery, the patient insisted on receiving the last injection and managed this without a syncope. All patients reported injections in the parotid glands to be particularly painful (CTCAE grade 1), but the pain quickly subsided after the procedure. One patient with hypertension and hypercholesteremia and 2 prior transient ischemic attacks encountered a stroke 82 days after the intervention, which the neurologists deemed unrelated to the intervention. No biochemical changes in parameters of liver and kidney function, blood count, or infection were observed during the 4 months follow-up. Four of the 10 patients were followed for at least 2 years with no treatment-related SAEs.

**Table 2.** Safety profile with adverse events.

Adverse events (AEs)	*N*	Sum	Proportion
Serious adverse events	10	1	0.1
SAEs, study related	10	0	0.0
SAEs, not related	10	1	0.1
Stroke			
Grade 1	10	0	0.0
Grade 2	10	0	0.0
Grade 3	10	1	0.1
Death	10	0	0.0
Adverse events, study related			
Pain at injection site	10	1	0.1
Grade 1	10	10	1.0
Grade 2	10	0	0.0
Grade 3	10	0	0.0
Syncope, vasovagal			
Grade 3	10	1	0.1
Adverse events, not study related^a^	10	3	0.3
Flu-like symptoms			
Grade 1	10	3	0.3
Grade 2	10	0	0.0
Grade 3	10	0	0.0

Not study-related adverse events are patient-reported all occurring between day 5 and 4 months.

AE, adverse event; SAE, serious adverse event.

**Figure 2.**
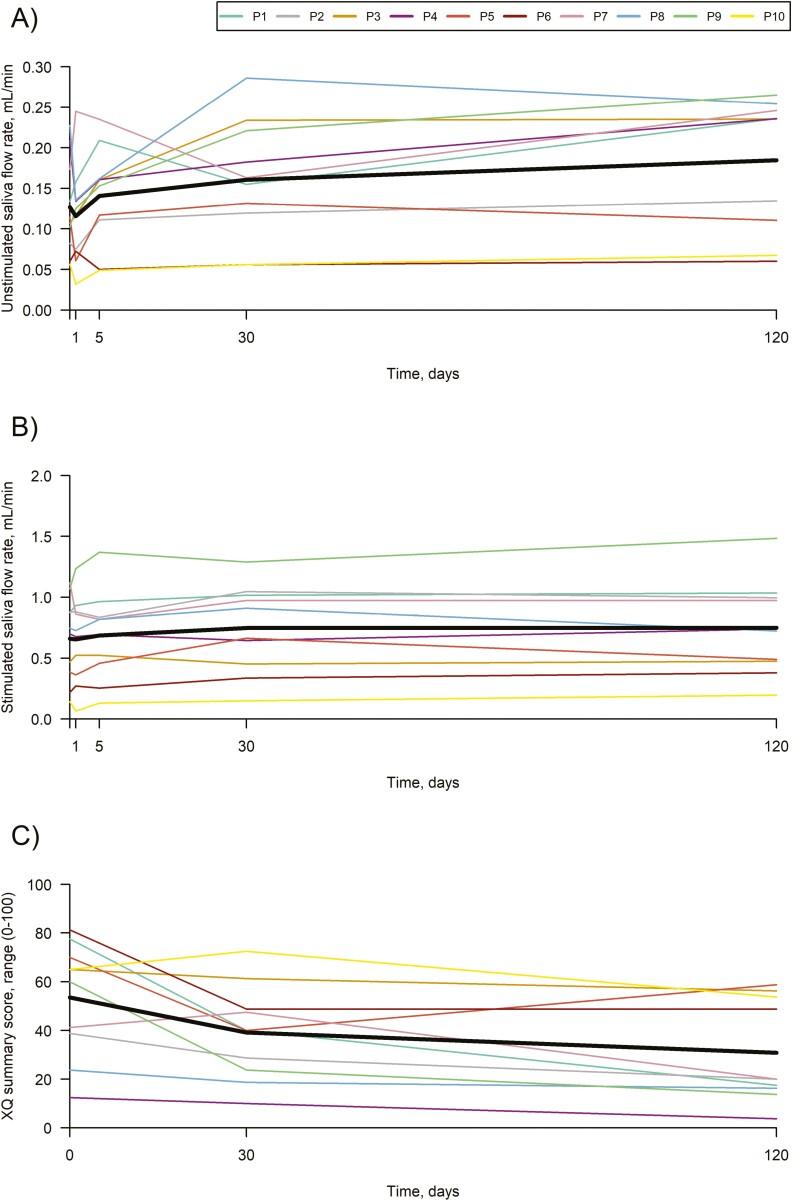
Unstimulated whole saliva flow rate over time (**A**), stimulated whole saliva flow rate over time (**B**), and Xerostomia questionnaire summary score over time (**C**). Lines with color represent the 10 patients and the black lines represent the least square means.

UWS flow rate increased from 0.13 mL/minute at baseline to 0.16 mL/minute after 1 month, corresponding to an increase of 0.03 mL/minute (95% CI: 0.00 to 0.07; *P =* .041). After 4 months UWS flow rate increased to 0.18 mL/minute, corresponding to an increase of 0.06 mL/minute (95% CI: 0.03 to 0.09; *P =* .0009) or 50% in UWS flow rate (95% CI: 24 to 76 *P* = .0005) from baseline (Table 3, Fig. 2A). SWS flow rate increased by 0.09 mL/minute (95% CI: 0.02 to 0.16; *P* =.019) after 1 month. After 4 months the SWS flow rate was increased by 0.09 mL/minute (95% CI: 0.02 to 0.16; *P* =.017) or 20% (95% CI: 7 to 33, *P* =.004) from baseline to 0.75 mL/minute (Fig. 2B). There was no difference between the patients treated with ASCs in DMSO 5% and DMSO 10% (*P* > .5). After 4 months 5 patients had a clinically relevant increase above 30% in UWS flow rate and 3 patients had an increase above 30% in SWS flow rate. The concentrations of UWS and SWS sodium, chloride, potassium and phosphate in the patients and healthy controls were comparable and within normal ranges at baseline (Table 1). A normal physiologic saliva flow rate dependence of inorganic ions was shown from UWS to SWS secretion in the patients at baseline and at 4 months. No changes were found in the concentrations of the inorganic ions in UWS and SWS at 4 months compared with baseline.

**Table 3.** Summary of key secondary outcome measures.

Functional outcomes	*n*	Baseline^a^	120 days follow-up^a^	Difference	95% CI	*P-*value
UWS FR, mL/minute	10	0.13 ± 0.02	0.18 ± 0.02	0.06	0.03 to 0.09	.0009
UWS FR, % change from baseline	10	0 ± 12.4	50.2 ± 12.4	50.2	23.97 to 76.44	.0005
SWS FR, mL/minute	10	0.66 ± 0.11	0.75 ± 0.1	0.09	0.02 to 0.16	.017
SWS FR, % change from baseline	10	0 ± 6.5	20.2 ± 6.5	20.2	5.14 to 35.22	.0099
XQ summary score (0-100)	10	53.5 ± 6.7	30.9 ± 6.7	−22.6	−33.57 to −11.68	.0004
EORTC QLQ-H&N35 (scores 0-100)						
HNDR	10	73.3 ± 7.2	46.7 ± 7.2	−26.7	−46.93 to −6.41	.013
HNSS	10	46.7 ± 8.3	23.3 ± 8.3	−23.3	−41.52 to −5.15	.015
HNSW	10	26.7 ± 4.8	16.7 ± 4.8	−10.0	−17.87 to −2.12	.016
Scintigraphic uptake score (scores 0-4)						
All glands	10	1.7 ± 0.2	1.8 ± 0.2	0.1	−0.06 to 0.16	.34
PG	10	2.3 ± 0.2	2.4 ± 0.2	0.1	−0.06 to 0.16	.34
SMG	10	0.9 ± 0.2	0.9 ± 0.2	−0.1	−0.16 to 0.06	.34
Scintigraphic excretion fraction, %						
All glands	10	53.3 ± 4.8	55.3 ± 4.8	2.0	−4.75 to 8.76	.52
PG	10	59.3 ± 6.0	60.8 ± 6.0	1.4	−6.85 to 9.94	.70
SMG	10	40.3 ± 5.0	44.3 ± 5.0	4.0	−4.16 to 12.06	.28
Saliva composition, mmol/L						
UWS chloride	8	21.7 ± 2.3	19.0 ± 2.4	−2.68	−7.46 to 2.09	.23
SWS chloride	9	26.5 ± 3.4	25.9 ± 3.4	−0.61	−3.41 to 2.18	.63
UWS phosphate	8	4.7 ± 0.4	4.4 ± 0.4	−0.28	−1.44 to 0.88	.55
SWS phosphate	9	3.8 ± 0.2	3.6 ± 0.2	−0.22	−0.88 to 0.44	.47
UWS potassium	8	19.1 ± 1.4	18.2 ± 1.6	−0.91	−4.57 to 2.74	.54
SWS potassium	9	19.4 ± 1.1	19.4 ± 1.2	0.02	−2.85 to 2.81	.99
UWS sodium	8	8.4 ± 1.4	7.2 ± 1.5	−1.17	−3.80 to 1.46	.32
SWS sodium	9	14.7 ± 3.3	15.7 ± 3.4	1.02	−3.57 to 5.62	.62

Values are least squares means and standard errors, unless otherwise stated.

The XQ summary score was reduced from 53.5 to 30.9 (change from baseline: −22.6 [95% CI: −33.6 to −11.7]; *P* = .0004) at 4 months after the intervention (Fig. 2C). The EORTC QLQ-H&N35 domains for dry mouth (HNDR) decreased from 73.3 to 46.7 (change from baseline: −26.7 [95% CI: −46.9 to −6.4]; *P* = .0013), sticky saliva (HNSS) from 46.7 to 23.3 (change from baseline: −23.3 [95% CI: −41.5 to −5.2]; *P* = .0015), and swallowing (HNSW) from 26.7 to 16.7 (change from baseline: −10.0 [95% CI: −17.9 to −2.1]; *P* = .0016) 4 months after the intervention (Table 3).

At baseline, only 1 patient had HLA antibodies, including DSAs, which showed slightly increased intensity (MFI values) at 1 month and decreasing at 4 months. Two patients (P5 and P10) developed de novo DSAs as well as other HLA antibodies. Two patients (P2 and P3) developed HLA antibodies of very low intensity but no DSAs. The specificity and MFI values are depicted in Table 4. At baseline, salivary gland tracer uptake on salivary gland scintigraphy was impaired in 1 or more glands in all patients. No change in tracer uptake was observed 4 months after the intervention (change from baseline: 0.1 [95% CI: −0.06 to 0.16]; *P* = .34), nor was any change observed in excretory gland capacity (excretion fraction) (2.0 [95% CI: −4.75 to 8.76]; *P* = .52).

**Table 4.** HLA classes I and II antibodies in patients.

Patient	Donor	Allele	Baseline MFI	1 month MFI	4 months MFI
1	1	—	0	0	0
2	2	—	0	0	0
3	2	—	0	0	0
4	3	—	0	0	0
5	3	B*40(60) B*40(61	0	17 756 16 724	3470 3031
6	1	A*02 C*02 DRB1*04	11 664 1515 4446	17 976 4003 3853	14 619 2329 5635
7	2	—	0		
8	1	—	0		
9	2	—	0		
10	1	A*02 A*23(9) A*24(9) B*44(12) B*45(12)	0	5056 1608 0 9813 12 815	11 242 1894 2381 10 175 11387

HLA typing for donor 1: A: *02, *24, B: *40, *44, C: *02, *03, DRB1: *01, *04, DQB1: *03, *05.

HLA typing for donor 2: A: *02, B: *13, *40, C: *03, *06, DRB1: *07, *13, DQB1: *02, *06.

HLA typing for donor 3: A: *01, B: *08, *40, C: *03, *07, DRB1: *13, DQB1: *06.

## Discussion

In this first-in-human trial, we report novel data on the safety of major SG injection with an “off-the-shelf” allogeneic AT-MSC therapy in patients with radiation-induced salivary gland hypofunction and xerostomia. Importantly, no patients experienced SAEs. These findings are in accordance with results from a randomized controlled trial from our group involving injections of autologous AT-MSCs in the submandibular glands wherein no patients developed SAEs.^21^ In the present trial, we included treatment of the parotid glands and advanced to allogeneic AT-MSCs, a clinically applicable therapy. Allogeneic cells come from young, healthy donors who have never had cancer or undergone radiation therapy or chemotherapy. Results indicate that MSC viability is affected by donor gender, age, comorbidities, and cisplatin.^30,31^ Moreover, “off-the-shelf” drugs require few donors and can be manufactured for numerous patients. Thus, fewer people would need to undergo surgery, and recipients of the AT-MSC drug would not be limited to patients living close to hospitals with GMP-approved stem cell facilities but could potentially benefit patients worldwide and be manufactured at much lower price than an autologous AT-MSC product. The study was designed as a safety study since it was the first time for a cryopreserved and allogeneic AT-MSC treatment of major SGs in humans. To investigate a potential safety concern of the excipient for this specific use, the trial was designed to start with CS5 (5% dimethyl sulfoxide, [DMSO]) for the first 5 patients and if no safety concerns were observed to continue with CS10 (10% DMSO) for the last 5 patients (Supplementary Material, eFig. 1). We found no safety concerns with the 2 concentrations, why we would prefer in the next phase II clinical trial to use CS10, since our data indicate that it is superior to CS5 for long-term preservation of the present high cell concentration.

As this was an open-label trial with no randomization, no conclusions can be drawn about the effect of implantation of allogeneic AT-MSCs in the major SGs, but the results can support our hypothesis of AT-MSCs being able to restore the function of radiation-damaged SGs. A significant increase in UWS flow rate was observed from 0.13 to 0.18 mL/minute, indicating that allogeneic AT-MSCs have the same potential as autologous AT-MSCs.^21^ The increase of 50% is highly likely to have a clinical benefit, although still below the levels in the healthy controls (UWS flow rate = 0.45 mL/minute). Interestingly, the SWS flow rates only increased by 20%. The lower increase in SWS flow rate probably has limited clinical relevance and indicating the irradiated parotid glands may be less responsive toward AT-MSCs treatment than submandibular glands. The significantly increased UWS and SWS flow rates at 4 months were not mirrored in the inorganic-ion concentrations. Likewise, the improvement of 50% in UWS is presumably too small to be reflected in the semi-quantitative evaluations of changes in the scintigrams.

MSCs have been regarded as hypoimmunogenic since they lack major histocompatibility complex (MHC)-II.^32–35^ Assessment of immunogenicity of allogeneic unmatched MSCs in clinical trials is sparse. The patients were treated with allogeneic AT-MSCs without matching or the exclusion of patients with DSAs and no immunosuppressive treatment. The AT-MSCs boosted preexisting DSAs in 1 patient and 2 patients had a broad range of de novo generated antibodies, including DSAs. This is in accordance with previous experience in patients with heart failure, where only a small transient increase in tissue-type antibodies was seen toward one of 3 donors without any clinical symptoms and influence on clinical efficacy.^23^ In our study, neither the patient with preexisting DSAs nor the 2 patients who developed DSAs had a significant increase in UWS flow rate after AT-MSC therapy. Thus, control of DSAs status prior to allogeneic AT-MSC therapy may be relevant but would not exclude the risk of de novo generation of DSAs. Possible association between effect and DSAs would need verification in larger studies as the lack of effect in the present study could also be explained by the very low residual saliva function in the 2 patients at baseline (UWS flow rate 0.05 mL/minute).

The answers from the EORTC QLQ-H&N35 and XQ indicate that patients did feel alleviation of xerostomia, which mirrors the increased UWS flow rates. Determining who will benefit from the treatment requires further investigation in a larger randomized controlled trial that focuses on safety, development of DSAs, baseline levels of saliva production, and time from radiation therapy to AT-MSC treatment.

## Limitations

To our knowledge, this is the first prospective study in human to evaluate the safety of an allogeneic AT-MSCs therapy injected in the parotid and submandibular glands in previous patients with HNC.

The study’s main limitation is the single-center, nonrandomized design with a small sample size; thus, any efficacy inference from this study cannot be claimed to be causal. The present study was primarily designed to determine the safety and feasibility of intraglandular injection of allogeneic AT-MSCs cryopreserved in DMSO either 5% or 10%. A future randomized trial should consider a placebo solution containing DSMO. Importantly, we found it unethical to inject a placebo with DMSO into the parotid gland near the facial nerve of a control group before we had the first safety results.

## Conclusions

This trial provides the first results of treatment with an “off-the-shelf” allogeneic AT-MSC drug in the submandibular and parotid glands of patients with radiation-induced salivary gland hypofunction and xerostomia with no SAEs. Increased UWS and SWS flow rates, and alleviation of xerostomia were observed. This may open a new frontier in providing treatments to minimize radiation-related morbidity after HNC treatment.

## Supplementary Material

szac011_suppl_Supplementary_Material_1

szac011_suppl_Supplementary_Material_2

szac011_suppl_Supplementary_Material_3

szac011_suppl_Supplementary_Material_4

szac011_suppl_Supplementary_Material_5

## Data Availability

The data contained in this article may not be shared publicly due to ethical/privacy reasons.
