# Supplementary material for: Intraglandular Off-the-Shelf Allogeneic Mesenchymal Stem Cell Treatment in Patients with Radiation-Induced Xerostomia: A Safety Study (MESRIX-II)
Source: Stem Cells Transl Med. 2022 Apr 18;11(5):478–89. doi: 10.1093/stcltm/szac011 (PMC9154319; doi:10.1093/stcltm/szac011)
Supplement: szac011_suppl_Supplementary_Material_1 [file szac011_suppl_supplementary_material_1.pdf]

STATISTICAL ANALYSIS PLAN

**Study Title**
A Phase I Open Label Study Evaluating the Safety and Feasibility of Allogeneic Mesenchymal stem cells for radiation-induced hyposalivation and xerostomia in previous head and neck cancer patients<br><br>Title on clinicaltrials.gov<br>Allogeneic Mesenchymal Stem Cells for Radiation-induced Hyposalivation and Xerostomia/Dry Mouth (MESRIX-SAFETY)
**Name of Experimental Intervention**
Intragländular Allogenic Adipose-derived Mesenchymal Stem/stromal Cells
**Study Number**
The National Committee on Health Research Ethics number: 1808924<br>**EudraCT:** 2018-003856-19<br>**Clinicaltrials.gov:** NCT03874572
**Protocol Version (Date)**
2.8 Marts 2020
**Analysis Type**
Longitudinal cohort study of 4 months (120 days) duration: Repeated measurements open label trial.
**Statistical Analysis Plan Version**
Version 1.0 (2020-nov-04)
**Statistical Analysis Plan Authors**
Robin Christensen, BSc, MSc, PhD; Professor of Biostatistics and Clinical Epidemiology; Department of Clinical Research, University of Southern Denmark, Odense University Hospital, Denmark.
Charlotte Lynggaard, MD, PhD Student; Chief investigator
Christian von Buchwald, MD, PhD; Trial Sponsor

ABBREVIATIONS

ASCs	Adipose-derived MSCs
CSCC_ASC	Cardiology Stem Cell Centre adipose-derived stromal cell
CTCAE	Common Terminology Criteria for Adverse Events
ENT	Ear-Nose-Throat
EORCT	European Organisation for Research and Treatment of Cancer
GCP	Good clinical practice
HRQoL	Health-related Quality of life
ICH	International Conference on Harmonization
ITT	Intention-to-treat
LPLV	Last patient last visit
LTFU	Long-term follow-up
MAR	Missing At Random
MCAR	Missing Completely At Random
MRI	Magnetic Resonance Imaging scan
MSCs	Mesenchymal stem/stromal cells
PROM	Patient-reported outcome measurements
QLQ	Quality of life Questionnaire
QoL	Quality of life
RCT	Randomized Clinical Trial
SAE	Serious adverse event
SAR	Serious adverse reaction
SAP	Statistical Analysis Plan
SG	Salivary gland
VAS	Visual-Analogue-Symptomatic scale
XQ	Xerostomia Questionnaire

1. INTRODUCTION

Background and rationale

The purpose of the current study is to assess the efficacy and safety of the injection of allogeneic adipose tissue-derived mesenchymal stromal/stem cells (MSCs) from healthy donors} on radiation-induced salivary gland hypofunction and xerostomia in patients with previous oropharyngeal cancer. The project can potentially help to develop a clinically relevant treatment option for the growing number of patients suffering from xerostomia after irradiation.

Study Objective(s)

The study objectives are to:

- to evaluate the safety and tolerability of treatment with allogeneic adipose tissue-derived MSCs transplanted into both submandibular and parotid glands in patients with radiation-induced hyposalivation and xerostomia.
- to evaluate the feasibility and efficacy of the treatment with allogeneic adipose tissue-derived MSCs transplanted into both submandibular and parotid glands assessed by change in salivary flow after one and four months compared to baseline in patients with radiation-induced hyposalivation and xerostomia.

2. STUDY METHODS

Trial Design

The study is an investigator-initiated, prospective, single-centre, uncontrolled, open-label trial with repeated measurements performed to investigate the safety and apparent efficacy of allogeneic adipose tissue-derived mesenchymal stem cells (ASCs) as a treatment for radiation-induced hyposalivation and xerostomia in a few highly motivated patients with previous oropharyngeal cancer patients. The intention-to-treat population will represent 10 patients with severe complications and reduced quality of life after radiotherapy. The study will evaluate the safety and efficacy of allogeneic adipose-tissue-derived MSCs study drug, CSCC(50). Because the allogeneic ASC product has not been tested in human salivary glands, this study is designed as an uncontrolled, open-label Phase I study.

The first 5 patients received the study drug with 5% DMSO and the last 5 patients received the study drug with DMSO 10%. The first two participants in each DMSO-group (patient 1-2 and patient 6-7) will at the intervention (baseline) have the ASCs transplanted into both submandibular glands and the right parotid gland, come again the following day checkup and blood samples. After five days the first two patients (patient 1-2 and 6-7) returned for checkup and have the ACSCs injected into the left parotid gland and left submandibular gland.

Randomization

N/A.

Intervention Model: Single group Assignment

Masking: None

Sample Size and Power

The sample size for this study was not determined by formal statistical methods but was based on extent and availability of data (i.e. feasibility).

Statistical Interim Analyses and Stopping Guidance

N/A

Timing of Final Analysis

The final analysis will be performed after last patient, last follow-visit (LPLV).

Timing of Outcome Assessments

3. STATISTICAL PRINCIPLES

Confidence intervals and *P* Values

All 95% confidence intervals (95%CI's) and *P* values will be two sided. We will not apply explicit adjustments for multiplicity, rather we will analyze and interpret these exploratory data with caution. Thus, we will disclose that our exploratory findings might reveal a false discovery (i.e. possibly having a false-positive inference); i.e. recalling that the significance level represents the risk of making a false discovery in any individual test.

Adherence and Protocol Deviations

All will be described

Analysis Populations

Full Analysis Set:

The analyses will be based on the Intention to Treat (ITT) population, i.e., based on the Full Analysis Set. This principle asserts the effect of the treatment that was planned, rather than the actual treatment given. Accordingly, participants included in the treatment group will be followed up, assessed and analyzed as members of that group, irrespective of their adherence to the planned course of treatment (i.e., independent of withdrawals, cross-over phenomena, and missing data in general).

Repeated Measurements Using Mixed Models: Our analyses will be based on the ITT population, including all included patients with available data at baseline. Missing data will be handled indirectly and statistically modeled using repeated-measures linear mixed models (see below). These models will be valid if data are 'Missing at Random' (MAR): i.e. "*Any systematic difference between the missing values and the observed values can be explained by differences in observed data*". Contrasts between different time points (i.e. within group analyses) will be estimated based on repeated-measures analysis mixed linear models (i.e., at t= 120 days from baseline considered the primary endpoint assessment).

As Observed Analysis Set:

For these analyses we will assume that the mechanism causing missing data may depend neither on observed data nor on the missing data; we refer to these analyses as being valid if data is missing completely at random (MCAR). We will report these with (basic) descriptive statistics (i.e. Medians and Interquartile ranges). If the MCAR assumption is valid, even an incomplete dataset will be representative for the entire dataset.<br>These patients could potentially be fundamentally different from those who were completing the entire 120 days with repeated visits. The characteristics that differ between patients who received the intended therapy and were assessed at the scheduled visits, and those who did not could easily influence whether the MESRIX intervention is a potentially doable treatment strategy.
Per Protocol Analysis Set:
The set of data generated by the subset of participants who complied with the protocol sufficiently to ensure that these data would be likely to exhibit the effects of treatment, according to the conceptual biomedical model. Compliance covers such considerations as exposure to treatment, availability of measurements and absence of major protocol violations.

4. TRIAL POPULATIONS

Screening Data

Information of the screening, eligibility and recruitment will be described in the paper and illustrated in the modified CONSORT flow diagram

Eligibility

Patients who had radiation-induced xerostomia and salivary gland dysfunction following radiotherapy for an oropharyngeal squamous cell carcinoma (OPSCC) and a minimum of two of follow-up without relapse were eligible for the trial. The full list of criteria is listed below.

Inclusion criteria

1. Age between 18-75 years
2. Previous radiotherapy +/- chemotherapy for OPSCC stage I- II (UICC-8, 2017).
3. 2 years' follow-up without recurrence
4. Clinically reduced salivation and hyposalivation, evaluated by a screening
5. Unstimulated salivary flow rate between 0.2mL/min and 0.05mL/min
6. Grade 1-3 xerostomia
7. WHO Performance status 0-12
8. Informed consent

Exclusion criteria

1. Any cancer in the previous 4 years (not including OPSCC and basocellular carcinomas)
2. Xerogenic medications
3. Penicillin or Streptomycin allergy
4. Any other diseases of the salivary glands, e.g. Sjogren's syndrome or sialolithiasis
5. Previous parotid or submandibular gland surgery
6. Previous treatment with any type of stem cells
7. Breastfeeding, pregnancy or planned pregnancy within the next 2 years
8. Smoking within the previous 6 months.
9. Alcohol abuse (consumption must not exceed 7 units/week for women and 14 units/week for men (Danish National board health alcohol guidelines3)

10. Any other disease/condition judged by the investigator to be grounds for exclusion

Controls

10 age-, sex-, race- and education matched controls were included to compare saliva samples. The controls had to fulfill the same inclusion and exclusion criteria as the patients apart from xerostomia and salivary gland dysfunction and previous OPSCC and radiotherapy.

Recruitment

The study participants were recruited through the ENT Department of Rigshospitalet and Oncology Departments at Rigshospitalet and Herlev Hospital.

Withdrawal/follow-up

The trial will be discontinued for individual participants in cases where they wish to be withdrawn from the current protocol or in exceptional circumstances where it is impossible to complete the experiment. Likewise, extraordinary events that render the project incomplete in its entirety will lead to withdrawal for all ongoing trials participants.

Baseline Patient Characteristics

Patients with prior p16+ or p16- oropharyngeal squamous cell carcinoma with radiation-induced xerostomia and dysfunction of the major saliva glands. Baseline characteristics will be presented in a table in the study publication.

5. ANALYSIS

Outcome Definitions

Primary Outcome Measures: From intervention to 4 months (120 days) follow-up

1. Safety: Number of patients with serious adverse events

   Registration of number of patients with serious adverse events in a 4 months follow-up period (collected at 0, 1, 5, 30, and 120 days from baseline)

Secondary Outcome Measures: From intervention to 4 months (120 days) follow-up

1. Immune response to treatment with allogeneic stem cells

   Registration of development of tissue antibodies towards donor cells (collected at 0, 30, and 120 days from baseline)

2. Efficacy: Change in unstimulated whole Salivary gland flow rate

   Unstimulated whole saliva flow rate is assessed by sialometry. (collected at 0, 1,5 30, and 120 days from baseline)

3. Efficacy: Change in stimulated whole Salivary gland flow rate

   Stimulated whole saliva flow rate is assessed by sialometry (collected at 0, 1,5,30, and 120 days from baseline)

4. Efficacy: Change in quality of life

   Quality of life evaluated by EORCT QLQ-H&N35 and XQ questionnaires (collected at 0, 30, and 120 days from baseline)

5. Efficacy: Salivary gland function

   Assessed by salivary gland 99mTc scintigraphy (performed at baseline and after 120 days from baseline)

6. Efficacy: Saliva composition analysis

   Saliva composition of inorganic ions (performed at baseline and after 120 days)

Analysis Methods

The objectives of repeated measures designs are to make inferences about the expected values of the observations, that is, about the means of the populations from which participants are

sampled. This objective is achieved by taking into account treatment and time effects in the model. Data will be analyzed using SAS, with the particular outcome variable (Yi), using a multilevel repeated measures random effects model with participants as the random effect factor based on a restricted maximum likelihood (REML) model.

For the continuous outcomes, these will be the response (dependent) variable, unstimulated and stimulated whole saliva flow rate and time (days; 5 levels) will be included as fixed effect covariates, as well as the interaction between treatment group and time; Patient ID will be handled as a random effects factor. This statistical model will hold all between-time comparisons for all assessment points up to 120 days from baseline (including baseline) and allows for evaluation of the average effect, as well as the trajectory over time from baseline to 120 days follow-up.

For the purpose of sensitivity, we will analyze the patients with missing data "as observed" comparing differences between baseline and 4-months Wilcoxon ranks test presented as Medians with interquartile ranges.

Missing Data and Outliers

The detection of outliers can be an important problem in model building, inference and analysis of a regression model like the linear mixed models we will apply. Since the presence of outliers can lead to biased estimation, misspecification of the model and inappropriate predictions.

We will apply model diagnostics (incl 'Cooks D'), in order to evaluate the distribution of studentized residuals and detect the presence of outliers and influential points; using studentized residuals and Cooks distance for detecting outliers in Y-direction plotted against the predicted estimates.

Strata and Covariates

Not applicable.

Additional Analyses

Harms

This will be assessed as elaborated on as part of the primary protocol; measuring Serious adverse event related to the intervention, Serious Adverse events, not related, and patient-reported adverse events.

**Statistical Software**
All statistical analyses will be performed in the software program SAS
**References**

CONFIDENTIAL

6. MANUSCRIPT OUTLINE

Figure 1. Flow diagram of patients assessed at each timepoint

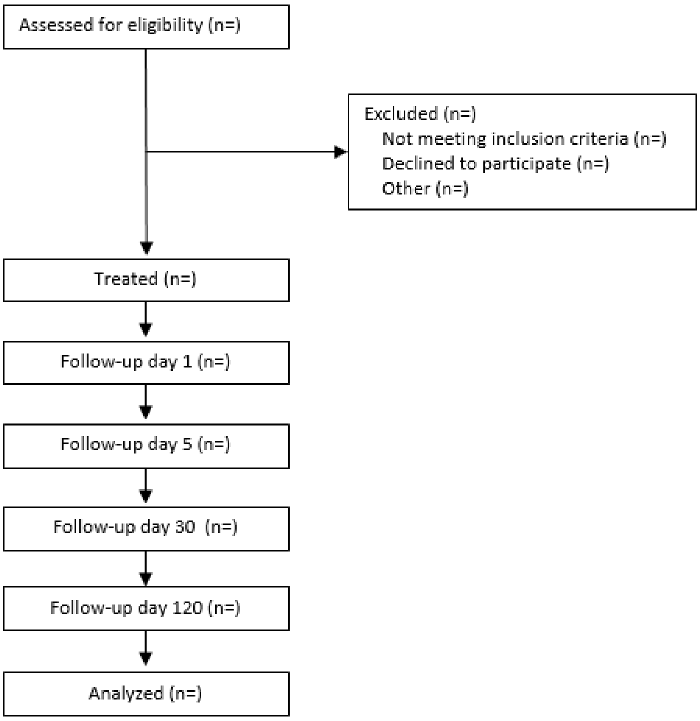

Table 1: Demographics and Baseline Characteristics

Table 1. Demographics of study participants

**Variables**	*Patients (n=10)*	*Controls (n=10)*
Age, years		
Sex, males, no. (%)		

Race, Caucasian, no. (%)
Donor specific antibodies
Education levels, no. (%):
Unskilled worker
Semi-skilled
Skilled worker
Bachelor
Master
DMSO high, no. (%)
Treatment over 2 days, no. (%)
Whole salivary flow rates mL/min:
Ustimulated whole saliva flow rate
Stimulated whole saliva flow rate
Saliva composition, mmol/l:
UWS Chloride
SWS Chloride
UWS Magnesium
SWS Magnesium
UWS Phosphorus
SWS Phosphorus
UWS Potassium
SWS Potassium
UWS Sodium
SWS Sodium
Primary tumor location, no. (%):
Tonsil
Base of tongue
p16+, no. (%)
Cancer stage (UICC 8):
1
2
IMRT + concurrent cisplatin, no. (%)
Radiation dose SMG:
Mean Gy right SMG ±SD
Max Gy right SMG ±SD
Mean Gy left SMG ±SD
Max Gy left SMG ±SD
Radiation dose PG:
Mean Gy right PG ±SD
Max Gy right PG ±SD
Mean Gy left PG ±SD
Max Gy left PG ±SD
Smoking status:
Never smoker
Previous 0-10 PY
Previous > 10 PY
Years from radiotherapy

CONFIDENTIAL

Table 2: Primary Endpoint

Table 2. Adverse events and donor specific antibodies

	n=10	%
Death		
Serious adverse events*		
Serious adverse event related to the intervention, no. (%)		
Serious Adverse events, not related, no. (%)		
Adverse events related to the intervention		
Other adverse events (PRO)		
Donor specific antibodies, no (%)		

Table 3: Key Secondary Endpoints

Functional Outcomes	*n*	Baseline	4-month follow-up	Difference	95% CI	*p* value
UWS FR, mL/min						
SWS FR, mL/min						
XQ-summary score (0-100)						
EORCT QLQ-H&N35 (score 0-100)						
HNDR						
HNSS						
HNSW						
Uptake score, (score 0-4):						
All glands						
PG						
SMG						
Excretion fraction, %						

All glands
PG
SMG
Saliva composition, mmol/l:
UWS Chloride
SWS Chloride
UWS Magnesium
SWS Magnesium
UWS Phosphorus
SWS Phosphorus
UWS Potassium
SWS Potassium
UWS Sodium
SWS Sodium

Figure 3

Figure 3.a (Spaghetti plot and plot with mean)

Unstimulated salivary flow rate

Figure 3.b

Stimulated salivary flow rate

Figure 3.c

XQ summary score

Appendix 1:

Study design

CONFIDENTIAL

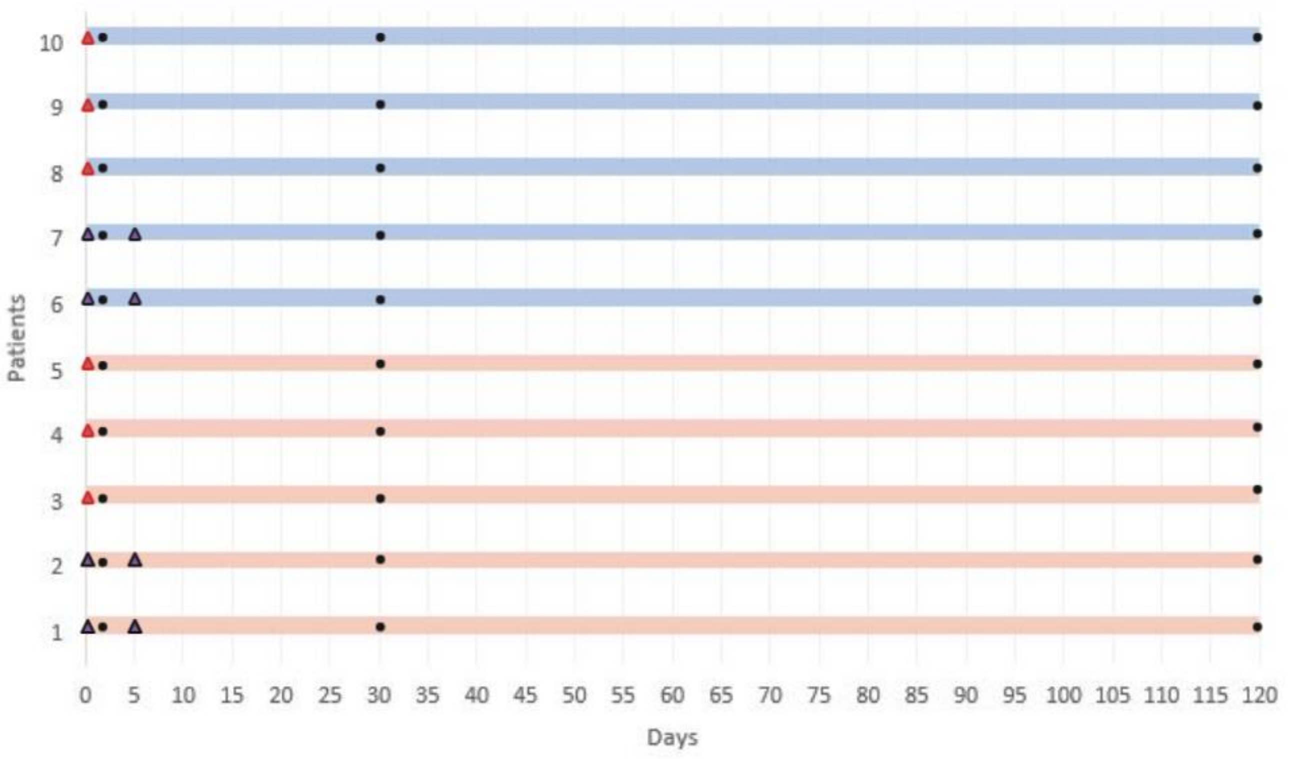

Appendix 2

HLA class I and II antibodies in patients

Patient	HLA typing	donor	Allele	Baseline	1 month	4 months
