# Supplementary material for: Intraglandular Off-the-Shelf Allogeneic Mesenchymal Stem Cell Treatment in Patients with Radiation-Induced Xerostomia: A Safety Study (MESRIX-II)
Source: Stem Cells Transl Med. 2022 Apr 18;11(5):478–89. doi: 10.1093/stcltm/szac011 (PMC9154319; doi:10.1093/stcltm/szac011)
Supplement: szac011_suppl_Supplementary_Material_2 [file szac011_suppl_supplementary_material_2.pdf]

Supplementary Online Content

This supplementary material has been provided by the authors to give readers additional information about their work.

eFigure 1. Study design

eFigure 2. Study flow chart

eMethods 1. Inclusion and exclusion criteria

eMethods 2. Salivary gland scintigraphy

eMethods 3. Inorganic salivary ions

eTable 1. Na^+ and K^+ concentrations in parts per billion obtained for 1643f standard by inductively coupled plasma mass spectrometry (ICP-MS) in this study compared to NIST certified composition*

eFigure 1. Study design.

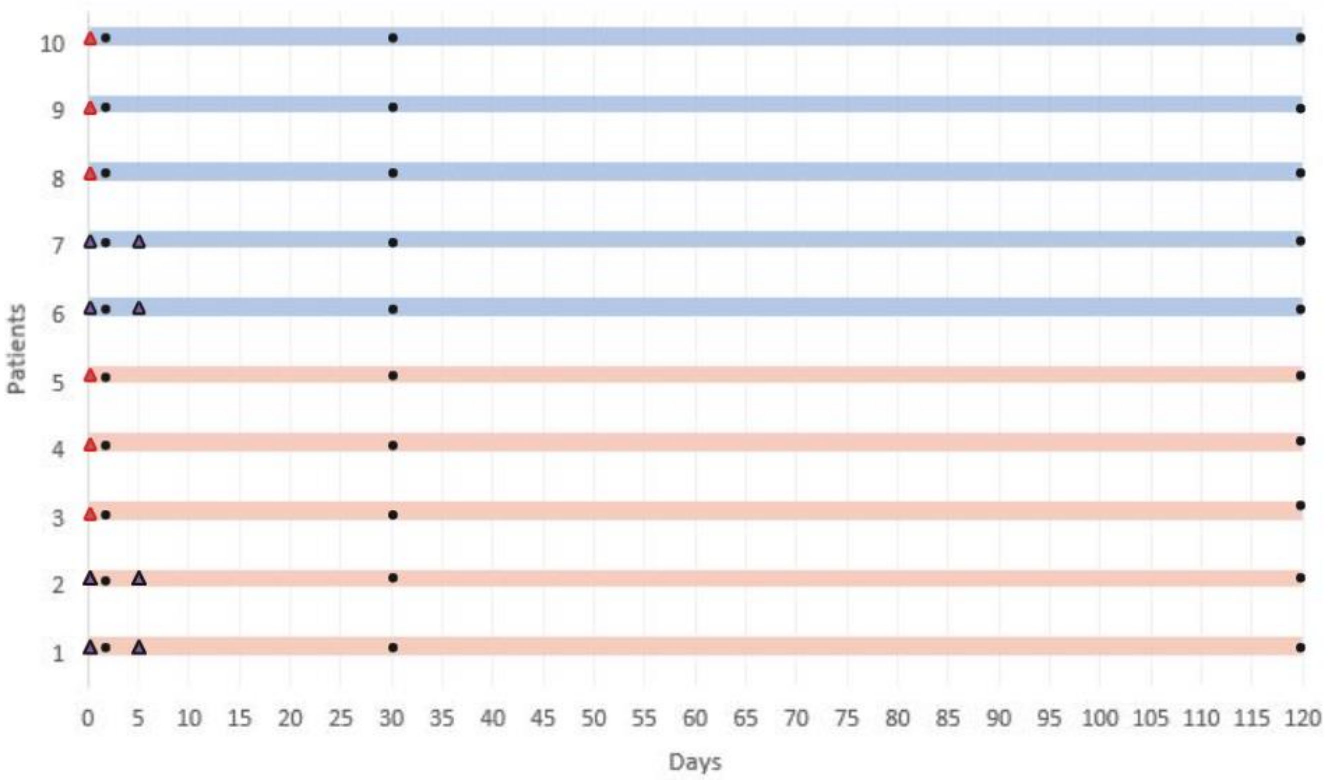

eFigure 1. The first 5 patients were treated with AT-MSCs cryopreserved in dimethyl sulfoxide (DMSO) 5% and the last 5 patients with AT-MSCs cryopreserved in DMSO 10%. Patients number 1 and 2 were treated only in the right salivary glands on day 0 and they came back to receive injection in the left glands on day 5. The treatment for patients number 6 and 7 was again divided on day 0 and day 5. The remaining 6 patients (3-5 and 8-10) had injection in all 4 glands on day 0. Created using Biorender.com (PL22Y4W85V).

eFigure 2. Study flow chart.

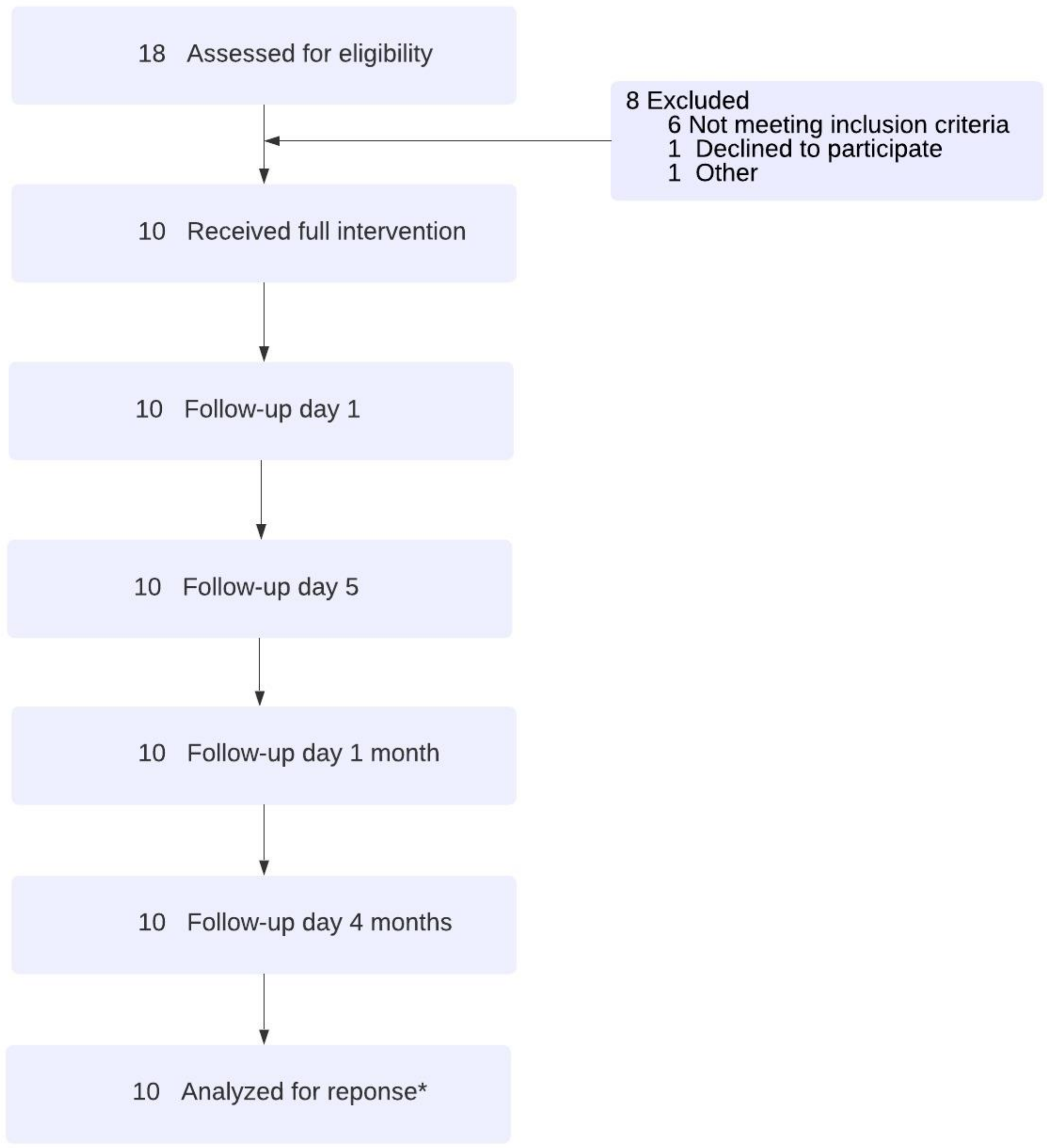

eMethods 1. Inclusion and exclusion criteria

Inclusion criteria

1. Age between 18-75 years
2. Previous radiotherapy +/- chemotherapy for OPSCC stage I-II (UICC-8, 2017).
3. 2 years' follow-up without recurrence
4. Clinically reduced salivation and salivary gland hypofunction, evaluated by a screening
5. Unstimulated whole saliva flow rate between 0.2mL/min and 0.05mL/min
6. Grade 1-3 xerostomia by CTCAEv5.0
7. WHO Performance status 0-2 (1)
8. Informed consent

Exclusion criteria

1. Any cancer in the previous 4 years (not including OPSCC and basal cell carcinomas)
2. Xerogenic medications
3. Penicillin or Streptomycin allergy
4. Any other diseases of the salivary glands, e.g., Sjögren's syndrome or sialolithiasis
5. Previous parotid or submandibular gland surgery
6. Previous treatment with any type of stem cells
7. Breastfeeding, pregnancy, or planned pregnancy within the next 2 years
8. Smoking within the previous 6 months.
9. Alcohol abuse (consumption must not exceed 7 units/week for women and 14 units/week for men (Danish National board health alcohol guidelines)[2]
10. Any other disease/condition judged by the investigator to be grounds for exclusion

eMethods 2. Salivary gland scintigraphy

Dynamic images were acquired on a single-head gamma camera Mediso TH45 (Budapest, Hungary) using a low-energy high-resolution parallel-hole collimator. The patient was placed in a supine position and an anterior head-neck projection was used. Images were recorded using a 64x64 pixel matrix for 30 min immediately following the intravenous injection of 250 MBq ^{99m}Tc-pertechnetate. Frame duration was 1 min, yielding a 30-frame dynamic study. After 20 min acquisition 3 mL lemon-juice was administered to the mouth without inducing head movement.

Saliva production, evaluated by radiotracer salivary-gland uptake, was accessed in a visual semi-quantitative setup scoring each parotid and submandibular gland. Dynamic cine and reframed 5 min images were inspected by 2 experienced nuclear medicine physicians (J.M., P.O.) using a salivary gland scintigraphy -dedicated software developed by Segami Oasis, incorporated in the IMPAX image system (ver. 6.7 AGFA Healthcare, Mortsel, Belgium). Each gland was scored using a 5-point system: 0) no activity above the background level; 1) severely decreased tracer uptake; 2) moderately decreased uptake; 3) mildly decreased uptake; and 4) normal tracer uptake.

Salivary gland radiotracer activity was quantitatively evaluated before and after lemon juice stimulation to assess/measure maximum saliva secretion. Regions of interest were drawn on the dynamic images, generating time-activity curves for each functioning gland. We recorded background corrected activity at max (c-max) and activity after the lemon juice-induced sharp drop-in activity (c-post). Salivary gland excretion fraction was calculated as EF (%) = 100 x (cmax – c-post) / cmax ([3]).

eMethods 3. Inorganic salivary ions

UWS and SWS samples were analyzed for sodium, chloride, potassium, and phosphate concentrations. UWS and SWS samples from patients and healthy controls were thawed and centrifuged at 15,550 x g for 20 min. The supernatant was taken to be filtered through a 0.2 µm syringe filter and this filtrate was diluted fivefold with deionized water and analyzed for chloride (Cl^-) and phosphate (PO_4^{3-}) concentrations in duplicate runs ([4,5]). Chromatographic measurement of chloride and phosphate concentrations was performed at Department of Dentistry and Oral Health, Aarhus University. Chromatographic separation was performed using ion chromatograph 761 Compact IC (Metrohm, Herisau, Switzerland), comprised of a high-pressure dual piston pump and a thermally controlled conductivity detector. Chloride and phosphate were determined on a Metrosep A Supp 5 – 250/4.0 (250 mm x 4 mm) anion separation column using 10 mM $NaHCO^3$ and 3.2 mM Na_2CO^3 eluent at 0.7 mL/min and suppressed conductivity detection. The chromatographic separation was achieved within 9 min for chloride and 20 min for phosphate. The sample injection volume was 20 µl. Calibration and linearity checks were performed with solutions of reagent-grade chemicals, Cl^- and PO_4^{3-} 1000 mg/L (Fisher Scientific, UK). Validation of the calibration and results was conducted by standard addition to the samples, in the context of eventual interference or matrix effect of human saliva, and recovery percentage was calculated ([4]).

Prior to analyses of sodium (Na^+) and potassium (K^+) concentrations by inductively coupled plasma mass spectrometry (ICP-MS), the UWS and SWS samples were centrifuged at 14,000 rpm for 20 min followed by sterilizing filtration to remove food remains, bacteria, and epithelial cells([5]). Around 0.1 mL of whole saliva was then transferred into acid-cleaned polypropylene tubes and mixed with 0.25 mL concentrated nitric acid (HNO_3), and 0.1 mL concentrated hydrochloric acid (HCl) and heated for 3 hours. Over the course of an hour, hydrogen peroxide (30% H_2O_2) was then repeatedly added (0.5 mL in total) to break organics until solutions cleared up and any film and/or coloring were gone. Prior to analysis, the samples were transferred to larger tubes and diluted 600 times with 3% v/v nitric acid.

Sodium and potassium concentrations were analyzed at Aarhus Geochemistry and Isotope Research (AGiR) Platform on an Agilent 7900 quadrupole ICP-MS run in helium gas mode to minimize interferences. Standard solutions prepared from SPEX CertiPrep ICP-MS Calibration Standard 3 were used to construct calibration curves by serial dilutions ranging from 10 ppb to 2 ppm. To monitor and correct for drift, a solution containing 100 ppb In (prepared from a 1000 mg\L Indium PlasmaCAL solution) was introduced as an internal standard during analysis by mixing it with the sample solutions via a peristaltic pump. All reagents used throughout were optima-grade distilled acids mixed with 18.2 MΩ water. Standards and blanks were run interspersed with sample solutions and the final analyses blank-corrected and reduced using Agilent software reduction package. Mean values of sodium and potassium concentrations obtained for National Institute of Standards and Technology (NIST, U.S. Department of Commerce) standard SRM (Standard Reference Materials) 1643f (trace elements in water; n=8) differed by 0% and 4% from certified values and reproducibility were 4% and 2% for sodium and potassium, respectively (see eTable 2 in Supplement 1 for details).

eTable 1. Na^+ and K^+ concentrations in parts per billion obtained for 1643f standard by inductively coupled plasma mass spectrometry (ICP-MS) in this study compared to NIST certified composition*

NIST 1643f - trace elements in water (n = 8)	Na^+ [ppb]	K^+ [ppb]
**Measured values in ppb**	18787	2006
**Standard deviation**	756	46
**Relative standard deviation**	4%	2%
**Certified values in ppb***	18830	1933
**Relative difference**	0%	-4%

*Certificate from National Institute of Standards and Technology (NIST), U.S. Department of Commerce, for Standard Reference Materials (SRM) 1643f - Trace Elements in Water.

References for Supplementary Online Content

1. Oken MM, Creech RH, Tormey DC, et al. Toxicity and response criteria of the Eastern Cooperative Oncology Group. *Am J Clin Oncol*. 1982;5(6):649-655. http://www.ncbi.nlm.nih.gov/pubmed/7165009. Accessed February 5, 2018.

2. DHMA. *Health Promotion Packages – Introduction and Recommendations*.; 2013.

3. Aung W, Murata Y, Ishida R, Takahashi Y, Okada N, Shibuya H. Study of quantitative oral radioactivity in salivary gland scintigraphy and determination of the clinical stage of Sjögren's syndrome. *J Nucl Med*. 2001;42(1):38-43.

4. Chen ZF, Darvell BW, Leung VWH. Validation of ion chromatography for human salivary anionic analysis. *Arch Oral Biol*. 2004;49(11):855-862. doi:10.1016/j.archoralbio.2004.05.001

5. Su Y xiong, Zhang K, Ke Z fu, Zheng G sen, Chu M, Liao G qing. Increased calcium and decreased magnesium and citrate concentrations of submandibular/sublingual saliva in sialolithiasis. *Arch Oral Biol*. 2010;55(1):15-20. doi:10.1016/j.archoralbio.2009.11.006
