# Supplementary material for: Intraglandular Off-the-Shelf Allogeneic Mesenchymal Stem Cell Treatment in Patients with Radiation-Induced Xerostomia: A Safety Study (MESRIX-II)
Source: Stem Cells Transl Med. 2022 Apr 18;11(5):478–89. doi: 10.1093/stcltm/szac011 (PMC9154319; doi:10.1093/stcltm/szac011)
Supplement: szac011_suppl_Supplementary_Material_5 [file szac011_suppl_supplementary_material_5.pdf]

# TITLE PAGE

## CLINICAL STUDY PROTOCOL

## A Phase I Open Label Study Evaluating the Safety and Feasibility of Allogeneic Mesenchymal stem cells for radiation-induced hyposalivation and xerostomia in previous head and neck cancer patients (MESRIX-SAFETY)

**Protocol code:** CVB2018-2
**EudraCT:** 2018-003856-19 **Clinical-Trials.gov:** NCT03874572
**The National Committee on Health Research Ethics number:** 1808924

**Sponsor** [a]Christian von Buchwald, MD, DMSc, Professor

**Principal investigator** [a]Charlotte Duch Lynggaard, MD, Ph.D.-student

**Co-investigators** [a]Christian Grønhøj Larsen, MD, Ph.D.

[b]Jens Kastrup, MD, MD, DMSc, FESC, Professor

[b]Annette Ekblond, MSc, Ph.D

[a]David Hebbelstrup Jensen, MD, Ph.D.

[c]Anne Fischer-Nielsen, MD, Ph.D.

[d]Jann Mortensen, MD, DMSc, Professor

[d]Peter Oturai, MD, senior consultant

[e]Lena Specht, MD, DMSc, Professor

The trial will be conducted according to the protocol and in compliance with Good Clinical Practice (GCP), the ethical principles stated in the Declaration of Helsinki, and other applicable regulatory requirements.

**Site of the clinical trial** [a]Department of Otorhinolaryngology and Audiology, Ear, Nose and Throat Surgery (ENT), 2071, Copenhagen University Hospital, Rigshospitalet Blegdamsvej 9, DK-2100 Copenhagen, Denmark

**English title:**
A Phase I Open Label Study Evaluating the Safety and Feasibility of Allogeneic Mesenchymal stem cells for radiation-induced hyposalivation and xerostomia in previous oropharyngeal cancer patients (MESRIX-SAFETY).

**Danish title:**
Et open-label studie med undersøgelser af sikkerhed og klinisk anvendelse af allogene mesenkymale stamceller til behandling af stråleinduceret hyposalivation og xerostomi hos patienter med tidligere oropharyngeal cancer.

**Danish layman title:**
Studie med undersøgelser af sikkerhed og anvendelse af donor stamceller til behandling af nedsat spytproduktion og mundtørhed efter strålebehandling for kræft i mundsvælget.

**APPROVALS**

The study protocol complies with the declaration of Helsinki, 64th WHA General Assembly, Fortaleza, Brazil, October 2013[1].

The protocol is to be approved by/ approval dates:

- The Danish Medicines Agency (DKMA/LMST)
- The Danish National Committee on Research Ethics (EC/NVK)
- The Danish Data Protection Agency (DPA, Datatilsynet)

**SITES OF SUPERVISORS**

[a] Department of Otorhinolaryngology and Audiology Ear, Nose and Throat Surgery (ENT), 2071, Rigshospitalet
[B] Cardiology Stem Cell Centre, The Heart Centre, Rigshospitalet
[c] The Cell Therapy Facility, The Blood bank, Dept. of Clinical Immunology, 2034, Rigshospitalet
[d] Department of Clinical Physiology, Nuclear Medicine and PET, 4011, Rigshospitalet
[e] Department of Oncology, 3994, Rigshospitalet

**APPENDICES**

| | |
|---|---|
| Appendix I: | Study flowchart |
| Appendix II: | Schedule of enrolment, interventions and assessments |
| Appendix III: | Schedule of long-term follow-up of Safety and Efficacy |
| Appendix IV: | Timetable |

## CLINICAL STUDY SYNOPSIS

**Protocol Title:** A Phase I Open Label Study Evaluating the Safety and Efficacy of Allogeneic Mesenchymal stem cells for radiation-induced hyposalivation and xerostomia in previous head and neck cancer patients (MESRIX-SAFETY)

**Sponsor**: Christian von Buchwald

**Protocol-Id:** CVB2018-2 **EudraCT number:** 2018-003856-19
**Name of the Active Ingredient:** Adipose-derived Allogeneic Mesenchymal Stem Cells
**Name of the Investigational Product:** Adipose-derived Allogeneic Mesenchymal Stem Cells for Xerostomia, Cardiology Stem Cell Centre Adipose tissue-derived Stromal/stem cell, CSCC_ASC(50)
**Study Phase:** 1
**Number of Investigational Centres Planned**: 1, Rigshospitalet, Copenhagen, Denmark
**Countries Planned:** 1, Denmark
**Planned Study Period**: 3Q/2018-4Q/2020 (last visit, last patient). Long-term follow will be performed under a follow-up protocol for a total of 5 years from the time of the transplantation.
**Number of Patients Planned**: 10 adults are planned to be enrolled. Replacement subjects may be added if subjects withdraw prior to conditioning.
**Study Population:** The study population will be composed of female and male patients with previous of head and neck squamous cell carcinoma stage I-II (UICC-8), age 18-70 years, inclusive, with radiation-induced hyposalivation and xerostomia with a minimum of two years of recurrence-free follow-up. The patients must be eligible for transplantation of allogeneic stem cells.
**Primary Objectives:**

- to evaluate the safety and tolerability of treatment with allogeneic adipose-derived mesenchymal stem cells CSCC_ASC(50) transplanted into both submandibular and parotid glands in patients with radiation-induced hyposalivation and xerostomia.
- to evaluate the efficacy of the treatment with allogeneic adipose-derived mesenchymal stem cells CSCC_ASC (50) transplanted into both submandibular and parotid glands assessed by change in salivary flow after one and four months compared to baseline in patients with radiation-induced hyposalivation and xerostomia.

**Study Endpoints**

*Safety Endpoints*

- Evaluation of the development of DSAs, incidence of SAEs and SUSARs of four months
- Immune response to allogeneic ASC. Measured by development of de novo human leucocyte antigen antibodies (HLA) and a diversity of immune mediated cytokines (an array of immune-related markers will be analyzed).

*Efficacy Endpoints*

- Change in salivary gland function measured by a four month change in unstimulated whole saliva flow rate.
- Change in quality of life. Evaluated by a change in complaints of xerostomia evaluated by two patient questionnaires (QLQ-H&N-35 and XQ).
- Change in saliva gland function assessed by salivary gland 99mTc scintigraphy after 4 months.
- Change in the composition (including RNA and proteomics) of saliva after one and four months, respectively

*Exploratory Endpoints*

- Correlation of efficacy and immune response

To evaluate the immune response to allogeneic ASCs. Measured by development of de novo human leucocyte antigen antibodies (HLA) and a diversity of immune mediated cytokines (an array of immune-related markers will be analysed)

- Patient-reported outcome measures: Change in quality of life. Evaluated by a change in complaints of xerostomia in the group receiving ASCs evaluated by two patient questionnaires (QLQ-H&N-35 and XQ)
- Change in saliva gland function assessed by salivary gland 99mTc scintigraphy after 4 months
- Change in the composition of saliva after one and four months, respectively

**General Design and Methodology:** The study is an investigator-initiated, prospective, non-randomized., open label, single-centre, single dose, Phase 1 study in 10 adults with prior oropharynx cancer and radiation-induced hyposalivation and xerostomia to receive transplantation of allogeneic mesenchymal stem cells into both parotid and submandibular glands. The patients will receive 50 million ASCs into both parotid glands and 25 million or 50 million ASCs in each submandibular gland according to the size of the glands. The trial will compare the safety, tolerability and efficacy of two different formulations of allogeneic adipose tissue-derived mesenchymal stromal/stem cells CSCC_ASC(50) as a treatment for radiation-induced hyposalivation and xerostomia for previous oropharyngeal cancer patients.
The study has three phases

- Pre-inclusion Screening, Informed Consent and Eligibility-determination Phase (Day -60-1)
- Intervention (Day 0)
- Follow-up (Day 1-4 months)

**Study Drug, Dose and Mode of Administration:** The study drug, allogeneic adipose tissue-derived mesenchymal stem cells, CSCC_ASC (50) will be injected ultrasound-guided intraglandular in both the parotid and submandibular glands. In our pilot study, MESRIX-I, the participants were randomised to placebo or a treatment of autologous adipose-derived mesenchymal stem cells with a cell dose of 2.8 x 106

ASC/cm3 gland, i.e., into both submandibular glands with no serious adverse events (EudraCT:2014-004349-29). Based on our experience from our previous study, the participant will receive 50 million ASCs into both parotid glands and 25 million or 50 million ASCs in each submandibular gland according to the size of the glands. This corresponds to an approximate total dose per patient at an average of $150x10^6$ – $200 \times 10^6$ ASCs when treating the four major salivary glands. The measurements of the salivary gland will rely on the size estimates by ultrasound combined with the measures from salivary gland scintigraphy. With ultrasound guidance, the intervention of ASCs will be transplanted into the four salivary glands. The first five patients will receive stem cells cryopreserved with DMSO 5 % and for the last five patients, the stem cells will have been cryopreserved in DMSO 10%.

**Investigational Products:** CSCC_ ASC(50)

**Duration of Patient Participation**: Patient participation will last for approximately 5-6 months (including a maximum of 60 day run-in-period and a 16 weeks follow-up period).

**Inclusion Criteria:**

1. Age between 18-75 years
2. Previous radiotherapy +/- chemotherapy for OPSCC stage I- II (UICC-8, 2017).
3. 2 years' follow-up without recurrence
4. Clinically reduced salivation and hyposalivation, evaluated by a screening
5. Unstimulated salivary flow rate between 0.2mL/min and 0.05mL/min
6. Grade 1-3 xerostomia as evaluated by CTCAEv5.0
7. WHO Performance status (PS) 0-1 [2]
8. Informed consent

**Exclusion Criteria:**

1. Any cancer in the previous 4 years (not including OPSCC and basocellular carcinomas)
2. Xerogenic medications
3. Penicillin or Streptomycin allergy
4. Any other diseases of the salivary glands, e.g. Sjögren's syndrome or sialolithiasis
5. Previous parotid or submandibular gland surgery
6. Previous treatment with any type of stem cells
7. Pregnancy or planned pregnancy within the next 2 years
8. Breastfeeding
9. Smoking within the previous 6 months.
10. Alcohol abuse (consumption must not exceed 7 units/week for women and 14 units/week for men (Danish National board health alcohol guidelines[3])
11. Any other disease/condition judged by the investigator to be grounds for exclusion

**Statistical Considerations and Sample Size Rationale:**
The sample size for this study was not determined by formal statistical methods but was based on extent and availability of data.

**Safety and Tolerability Analysis**: All adverse events are monitored at the scheduled follow-up (day one, 1 month and 4 months after the intervention). AEs will be assessed and graded according to Common Terminology Criteria for Adverse Events v5.0 guidelines (CTCAEv5.0).

**Efficacy Analysis:** The most common method for evaluating the salivary gland function and salivary flow rate is sialometry (salivary output measurement). A change in the secretion rate of the unstimulated whole saliva in the oral cavity is probably the most deciding parameter for the biological development of hyposalivation and xerostomia.

<u>The key efficacy endpoint</u>: The results on salivary flow rate will be calculated as a percentage change in salivary flow rate (from baseline) in the group of participants given ASCs

**Compensation:** Study participants will not be offered financial compensation will be offered to participate

**Data Monitoring Unit**: The trial will be conducted according to the Good Clinical Practice (GCP) guidelines and monitored by the GCP unit at the University of Copenhagen. The project will be carried out in accordance with the protocol, GCP guidelines and current Danish legislation. The project may be evaluated by an external audit.

**Site of the clinical trial**: Department of Otorhinolaryngology and Audiology, Ear, Nose and Throat Surgery (ENT), 2071, Copenhagen University Hospital, Rigshospitalet, Blegdamsvej 9, DK-2100 Copenhagen, Denmark

**TABLE OF CONTENTS**

TITLE PAGE 1

CLINICAL STUDY SYNOPSIS 3

*EFFICACY ENDPOINTS* 4
*EXPLORATORY ENDPOINTS* 4

LIST OF TABLES 10

LIST OF FIGURES 10

ABBREVIATIONS 11

1.BACKGROUND AND STUDY RATIONALE 14

1.1 STUDY RATIONAL 14

1.2 OVERVIEW OF RADIATION-INDUCED HYPOSALIVATION AND XEROSTOMIA 14
1.2.1 THE MAJOR SALIVARY GLANDS AND THE DAMAGE FROM RADIATION 15
1.2.2 HYPOSALIVATION, XEROSTOMIA AND COMPLICATIONS 15

1.3 OVERVIEW OF STEM CELL TREATMENT 16

1.3.1 MESENCHYMAL STEM CELLS AND ADIPOSE DERIVED-STEM CELLS 16
1.3.2 PRECLINICAL STUDIES: MESENCHYMAL STEM CELLS IN THE TREATMENT OF XEROSTOMIA 17
1.3.3 CLINICAL STUDIES AND OUR EXPERIENCE WITH MESENCHYMAL STEM CELLS FOR XEROSTOMIA 18

1.4 ALLOGENEIC STEM CELLS 19

1.4.1 RATIONALE FOR SHIFTING FROM AUTOLOGOUS TO ALLOGENEIC ASCS 19
1.4.2 SAFETY OF ALLOGENEIC MESENCHYMAL STEM CELLS 20
1.4.3 EFFICACY OF ALLOGENEIC MESENCHYMAL STEM CELLS VERSUS AUTOLOGOUS 21

2. RESEARCH HYPOTHESIS 23

3. STUDY OBJECTIVES AND ENDPOINTS 23

3.1 STUDY OBJECTIVES 23

3.2 STUDY ENDPOINTS 23

3.2.1 SAFETY ENDPOINTS 23
3.2.2 EFFICACY ENDPOINTS 23
3.2.3 EXPLORATORY ENDPOINTS 23

4. INVESTIGONAL PLAN 24

4.1 OVERALL DESIGN AND PLAN OF THE STUDY 24

4.1.1 STEP-BY-STEP DESCRIPTION OF THE STUDY 24

5. STUDY POPULATION 26

5.1 JUSTIFICATION OF STUDY POPULATION AND NUMBER OF PARTICIPANTS 26

5.2 INCLUSION CRITERIA 26

5.3 EXCLUSION CRITERIA 26

5.4 CRITERIA FOR WITHDRAWAL FROM THE STUDY 27

5.4.1 CRITERIA FOR WITHDRAWAL FROM THE STUDY BEFORE TREATMENT 27

5.5 CONTRACEPTION 27

6. METHODS 28

6.1 THE DOSE OF THE STUDY DRUG 28

6.3 TECHNIQUE FOR INTRAGLANDULAR ADMINISTRATION OF ASCS INTO THE SALIVARY GLANDS 28

7. STUDY ASSESSMENTS 29

7.1 ASSESSMENTS OF SAFETY 29

7.1.1 EVALUATION, REPORTING AND RECORDING OF ADVERSE EVENTS 29
7.1.2 TABLE 1: ADVERSE EVENTS WITH POSSIBLE RELATION TO INJECTION OF ASCS AND FNA 30

7.2 LONG-TERM SAFETY AND EFFICACY FOLLOW-UP 31

7.3 ASSESSMENTS OF EFFICACY 31

7.3.1 SIALOMETRY 31
7.3.2 SALIVARY GLAND SCINTIGRAPHY 32
7.3.3 ASSESSMENT OF THE QUALITY OF THE SALIVA: ANALYSIS OF SALIVA 32

7.4 PATIENT-REPORTED OUTCOME: METHODS FOR EVALUATING XEROSTOMIA 33

8. DATA ANALYSES 34

9. BIOBANKS RELATED TO THE STUDY 34

10. ETHICAL CONSIDERATIONS 34

11. RISK ASSESSMENT AND BENEFITS 35

11.1 THE PROCEDURE 35

11.2 MESENCHYMAL/ADIPOSE-DERIVED MESENCHYMAL STEM CELLS 35

11.3 THE STUDY DRUG 35

11.4 SCINTIGRAPHY 37

11.5 OVERALL RISK 37

11.6 BENEFITS OF THE PARTICIPANTS 38

12. DATA COLLECTION 39

13. RECRUITMENT OF PARTICIPANTS AND OBTAINING INFORMED CONSENT 40

13.1 GUIDELINES FOR SUBMISSION OF THE ORAL INFORMATION AND OBTAINING CONSENT 40

13.1.1 RECRUITMENT OF PARTICIPANTS AND OBTAINING INFORMED CONSENT 40

14. SUBJECT CONFIDENTIALLY 41

15. OPERATING AND FINANCIAL MATTERS 41

15.1 COMPENSATION 41

16. CHANGES TO PROTOCOL 41

17. PROPOSED DURATION OF RESEARCH AND DISSEMINATION PLAN 41

17.1 LOCATION AND TIMING OF STUDY 41

17.2 RESEARCH RESULTS 42

18. THE AVAILABILITY OF INFORMATION FOR PARTICIPANTS 42

19. DISCONTINUATION OF STUDY 43

20. INSURANCE 43

21. APPENDICES 44

APPENDIX II: TABLE 2: SCHEDULE OF ENROLMENT, INTERVENTIONS AND ASSESSMENTS 45
APPENDIX III: TABLE 3: SCHEDULE OF LONG-TERM SAFETY AND EFFICACY FOLLOW-UP (S&E-FOLLOW-UP) 46
APPENDIX IV: TABLE 4: TIMETABLE 47

22. REFERENCES 48

## LIST OF TABLES

Table 1: Reference safety information (RSI)

Table 2: Schedule of enrolment, interventions and assessments

Table 3: Schedule of long -term Safety and Efficacy follow-up (S&E-Follow-up)

Table 4: Timetable

## LIST OF FIGURES

Figure 1: Study flowchart

| ABBREVIATIONS | Definition |
|---|---|
| Ab | Antibody |
| BM | Bone Marrow |
| AE | Adverse event |
| aGVHD | Acute graft-versus-host disease |
| Anti-HBc | Hepatitis B core antibodies |
| Anti-HCV | Hepatitis C antibodies |
| AR | Adverse reaction |
| ASC | Adipose-derived stem cell |
| ASTRO | American Society for Radiation Oncology |
| ATIMP | Advanced therapy investigational medical product |
| BMI | Body mass index |
| BM-MSC | Bone Marrow-derived mesenchymal stem cells |
| CDMP | Clinical Data Management Plan |
| COSORT | Consolidated Standards of Reporting Trials |
| CPA | Cryoprotective Agent |
| CRF | Case Report Form |
| CTCAE | Common Terminology Criteria for Adverse Events |
| CTFG | Clinical trials facilitation group |
| $DMEM^{pHPL}$ | Dulbecco Modified Eagle Medium |
| DKMA | Danish Medicines Agency |
| DMC | Data Monitoring Committee |
| DMSO | Dimethyl sulfoxide |
| DNA | Deoxyribonucleic acid |
| DPA | Danish Data Protection Agency |
| DPBS | Dulbecco's phosphate-buffered saline |
| DSA | Donor –specific antibodies |
| DSM | Data and safety monitoring |
| DSUR | Development Safety Update Report |
| EC | National Committee on Health Research Ethics |
| EDC | Electronic Data Capture System |
| EGF | Epidermal growth factor |
| ELISA | Enzyme-linked immunosorbent assay |
| ENT | Ear, Nose and Throat |
| EORTC | European Organisation for Research and Treatment of Cancer |
| EOL | End of life |
| EOT | End of trial |
| ESC | Embryonic stem cell |
| EudraCT | European Clinical Trials Database |
| FSH | Follicle stimulating |
| GCP | Good Clinical Practice |
| GDPR | General Data Protection Regulation |
| GMP | Good Manufacturing Practice |
| GVHD | Graft-versus-host disease |
| HA | Human albumin |

| | |
|---|---|
| HBsAg | Surface antigen of the Hepatitis B virus/ Australia antigen |
| HBSS | Hanks balanced salt solution |
| HCG | Human chorionic gonadotropin |
| HCV | Hepatitis C virus |
| HIV-1 | Human immunodeficiency virus type 1 |
| HIV-2 | Human immunodeficiency virus type 2 |
| HLA | Human leucocyte antigen |
| HMA | Head of Medicines Agencies |
| HNC | Head and Neck Cancer |
| H&N | Head and Neck |
| HRQOL | Health- related quality of life |
| ICF | Informed Consent Form |
| ICH | International Conference on Harmonisation |
| ICMJE | International Committee of Medical Journal Editors |
| IHD | Ischaemic heart disease |
| IHF | Ischaemic heart failure |
| IMP | Investigational medical product |
| IMPD | Investigational medical product dossier |
| IMRT | Intensity-Modulated Radiation therapy |
| ISCT | International society for cellular therapy |
| iPS | Induced pluripotent stem cells |
| ITT | Intention-to-treat |
| IUD | Intrauterine device |
| IUS | Intrauterine hormone-releasing system |
| IV | Intravenous |
| Kg | Kilogram |
| LMST | Lægemiddelstyrelsen |
| LPLV | Last patient, last visit |
| LTFU | Long-term follow-up |
| MA | Maximum accumulation |
| MBq | Megabecquerel |
| MHC | Major histocompatibility complex |
| mL | Milliliter |
| MRI | Magnetic resonance imaging |
| MS | Maximum secretion |
| MSC | Mesenchymal stem cell |
| mSv | Millisievert |
| Na | Sodium |
| NaCL | Sodium chloride |
| NIDCM | Non-ischemic dilated cardiomyopathy |
| NVK | National Videnskabsetisk komite |
| OPSCC | Oropharynx squamous cell carcinoma |
| PCR | Polymerase chain reaction |
| PCT | Participation in clinical trial |
| PET | Positron emission tomography |

| | |
|---|---|
| pHPL | Human Platelet Lysate |
| PRO | Patient-reported outcome |
| PROM | Patient-reported outcome measure |
| PRP | Platelet-rich plasma |
| PS | WHO Performance Status |
| RCT | Randomised clinical trial |
| RIX | Radiation-induced Xerostomia |
| RNA | RiboNucleic Acid |
| RSI | Reference Safety Information |
| RT | Radiotherapy |
| QoL | Quality of life |
| QLQ | Quality of life Questionnaire |
| SAE | Serious adverse event |
| SAR | Serious adverse reaction |
| SAS | Statistical analysis software |
| SD | Standard Deviation |
| S&E | Safety and efficacy |
| SFR | Saliva flow rate |
| SG | Salivary gland |
| SPIRIT | Standard Protocol Items: Recommendations for Interventional Trials |
| SOP | Standard Operating Procedure |
| SPSS | Statistical package for the social sciences |
| SUSAR | Suspected unexpected serious adverse reactions |
| SVF | Stromal vascular fraction |
| Tc | Technetium |
| TESI | Transendocardial stem cell injection |
| UICC | Union for International Cancer Control |
| UR | Uptake ratio |
| USB | Universal Serial Bus |
| WHO | World Health Organisation |
| XQ | Xerostomia Questionnaire |

# 1.BACKGROUND AND STUDY RATIONALE

## 1.1 Study rational

The current study purpose is to assess the efficacy and safety of the injection of allogeneic adipose tissue-derived stromal/stem cells CSCC_ASC(50) from healthy donors on radiation-induced salivary gland hypofunction and xerostomia in patients with previous oropharyngeal cancer. The project can potentially help to develop a clinically relevant treatment option for the growing number of patients suffering from xerostomia after irradiation. The development of new therapies is especially meaningful since only suboptimal, symptomatic treatments are currently available, and since the symptom of xerostomia immensely reduces quality of life.

## 1.2 Overview of radiation-induced hyposalivation and xerostomia

The incidence of head and neck cancer is increasing in the Western World, including Denmark [4,5]. Cancer of the head and neck and its treatment often have a detrimental and lifelong impact on the quality of life of the patients [6]. Radiotherapy serve as a key component for approximately 80 % of all patients with head and neck cancer either as a single modality or in combination with chemotherapy and/or surgery[7]. Despite the enormous improvement in radiotherapy (IMRT, Intensity-Modulated radiation therapy), the treatment still inevitably leads to significant ionizing of healthy tissue, including the radiation-sensitive salivary glands. Damage of the major salivary glands results in hyposalivation and to a devastating dry mouth syndrome, xerostomia, for 74-85% of radiated head and neck cancer patients[7,8]. Xerostomia is not life-threating but predispose the patients to a wide range of debilitating oral disorders and have a major implication for the overall quality of life, including social life and professional life. Currently, only symptomatic treatment is available to patients suffering from xerostomia, therefore there is an prodigious unmet need for new treatment strategies [8,9].

We have recently completed an encouraging randomized-controlled pilot study with 30 patients assessing the safety and efficacy of ex-vivo expanded autologous adipose-derived mesenchymal stem cells for radiation-induced xerostomia in an effort to regenerate the function of the salivary glands (**EudraCT no:2014-004349-29**)[10,11]. The results of this study show most importantly that the stem cell treatment is safe without any serious adverse events and no systemic reactions, and secondly the patients treated with stem cells have a promising enhanced production of saliva of 33%-50%[11]. Further, the study showed that the stem cell-treated group gained vital quality of life measures compared with the placebo group in the form of diminished trouble in eating.

We here present a solid plan to continue our research in collaboration with the Cardiology Stem Cell Centre, Rigshospitalet with a phase I trial assessing the effect of an allogeneic adipose-derived mesenchymal "off-the-self-drug" for radiation-induced xerostomia in patients with previous oropharyngeal cancer and treat both the parotid and submandibular glands. The drug we wish to test is CSCC_ASC(50) which already tested safe so inject intramyocardially in 135 of patients with ischaemic heart disease (IHD) and ischaemic heart failure (IHF). CSCC_ASC(50) is based on the product CSCC_ASC already proven safe in a phase 1 safety trial for ischemic heart disease (CSCC_ASC safety study, EudraCT 2014-002980-13. Published: Stem Cells Transl Med. 2017 Nov;6(11):1963-1971). CSCC_ASC is currently being tested in two phase II clinical multicentre studies: The Copenhagen HF II study (EudraCT 2015-001560-19) and the SCIENCE Trial (EudraCT

No: 2015-002929-19). With more than 135 patients currently treated in these studies, no treatment related serious or non-serious adverse events, have been detected. CSCC_ASC is formulated holding 10% DMSO. CSCC_ASC(50) is a concentrated version of CSCC_ASC, holding 50 million ASCs per mL (in comparison to 22 million/mL) developed to meet treatment specific dosage needs for the indication radiation-induced hyposalivation.
We hope the results from this safety study will serve as a steppingstone and herald the way for a clinically relevant treatment to ameliorate patients with this severely hampering condition. If the present study confirms a clinically significant effect of mesenchymal stem cells and – as we have shown – remains safe, patients suffering from radiation-induced xerostomia could be offered a simple, minimally invasive procedure with few adverse events and risks, which contrasts with the current sub-optimal treatments.

### 1.2.1 The major salivary glands and the damage from radiation

The paired major salivary glands (SG) consist of the parotid, submandibular and sublingual glands, which account for 90% of the human production of 500-1500 mL/ day of the unique biologic fluid, saliva [12,13]. The major SGs have an elegant architecture of epithelial acini, connecting branching structures, vascular and neuronal networks that function together to produce and secrete saliva. The epithelial compartment of SGs consists of nearly 80% saliva secreting acinar and 20% saliva transporting/modifying ductal cells.
The acinar cells in particularly suffer severe damage from radiation. The molecular mechanisms underlying the radiation damages of the SGs are not fully understood, however the RT induce both qualitative and qualitative changes in the saliva. It is believed that the hypofunction of the glands is a result of acinar epithelial cell loss as well as damage to surrounding blood vessels, nerves and depletion of the stem cell reservoir in the salivary glands [14,15] . Thus, the radiation damage is partly elicited on the salivary gland stem cells, the healing and natural salivary gland regeneration is deterred. Hence, treatment with stem cells may be promising in restoring the function of the SGs. The submandibular glands contribute approximately two-thirds of unstimulated saliva volume, whereas parotid glands contribute with a mixed, mainly serous type of saliva during stimulation[16,14]. Studies indicate that the parotid glands are most sensitive to radiation and have the largest contribution during eating (stimulation)[16].

### 1.2.2 Hyposalivation, xerostomia and complications

The accepted range of normal flow for unstimulated saliva is anything above 0.1 mL/min, though there is a great variability in individual salivary flow rates. If individualized base rates have been established, then a 50 % reduction in flow should be considered hypofunction[13]. Xerostomia is a term describing the subjective feeling of dry mouth. Xerostomia can coexist with or exist without a reduced production of saliva, although xerostomia is first perceived when unstimulated whole saliva flow rate is reduced by more than 40-50% [17]. The two main causes of grave xerostomia are Sjögren's syndrome and radiation therapy for head and neck cancer, other causes are xerogenic medications, side effects from chemotherapy, rheumatic and dysmetabolic diseases.

The decrease of saliva secretion after radiotherapy predisposes the patients to a variety of conditions. These are either directly or indirectly a result of the decreased production of saliva and include xerostomia, impairment of taste perception and the normal oral functions (talking, chewing and swallowing) due to insufficient wetting. The reduced lubrication of mucosal surfaces makes the oral the oral mucosa vulnerable, which may lead to frictional trauma and ulceration and impairs the ingested food. In addition, a reduced salivary flow results in a reduced "rinsing" of the entire oral cavity, leading to microbial overgrowth, which in addition to other factors may result in rapid dental decay, dental erosion and oral candidiasis (thrush)[16,18].
Currently prophylactic use of amifostine to prevent xerostomia and the vast number of unsatisfying strategies to improve salivary gland function after radiation therapy have not met the need of patients. The symptomatic treatments are indeed limited and include pharmacological agents, for example sialogogues (e.g., pilocarpine, cevimeline, etc.), saliva stimulants (lozenges, gum and custom-made sour candy), and the use of oral lubricants and saliva substitutes.

## 1.3 Overview of Stem cell treatment

Stem cells have been identified as a potential treatment modality for a wide variety of disorders by their ability to differentiate into many functional cell types. It is widely accepted that stem cells can be divided into two major groups: Embryonic stem cells (ESCs) and non-embryonic stem cells[19]. Most of the early work on stem cells was performed in the pluripotent ESCs, which are derived from the inner cell mass of a blastocyst embryo (an early stage of pre-implanted embryo) and can differentiate into cells of all three primary germ layers. The clinical use of these cells as therapeutic agents is currently very limited, however, due to histocompatibility problems, their potential ability to form teratomas, and controversies over the ethics of using cells from human embryos. Difficulties with histocompatibility have partly been improved with the development of induced pluripotent stem cells (iPS cells), but many challenges lie ahead before iPS are an option for therapeutic therapy. Therefor non-embryonic stem cells, mostly adult stem cells, are highly attractive in the scientific field. Adult stem cells are defined as multipotent with a limited differentiation potential, i.e. with a narrower differential ability. Currently, the most commonly investigated adult stem cells in regenerative medicine are the mesenchymal stem cells (MSCs) [19] and as of May 23, 2018, there were 53,833 references in Pubmed to “mesenchymal stem cell” and 53,141 to “mesenchymal stem cells” .

### 1.3.1 Mesenchymal Stem Cells and Adipose Derived-Stem cells

MSCs are adult fibroblast-like stem cells, which reside in most tissues in the body, including the major salivary gland. The MSCs are best characterized and described in the bone marrow, where they have a presumed supportive function for haematopoiesis. The mesenchymal were initially presented more than 40 years ago by Friedenstein and colleagues from mice bone marrow and later referred to as mesenchymal stem cells by Caplan [20,21] .These cells constitute a population of

perivascular pericytes adjacent to small vessels being involved in local inflammation homeostasis and tissue replenishment[22]. Currently, there is no specific marker for MSCs. The International Society of Cellular Therapy (ISCT) has in 2006 recommended a standard set of minimal criteria for defining MSCs as follows: adherence to a plastic culture surface; expression of the surface markers CD73, CD90, CD105 and absence of surface markers CD14, CD34, CD45 (primarily haematopoietic); and the potential under appropriate in vitro conditions to differentiate more specialized cell types of the mesodermal germ layers (including adipocytes, myocytes, chondroblasts and osteoblasts) when subjected to the proper inductive factors and culturing media[23–25]. The first clinical studies have been conducted with bone-marrow MSC (BM-MSCs) however, adipose derived mesenchymal stem cells (ASCs) have several advantages compared to BM-ASCs as a higher yield of ASCs can be isolated from abdominal adipose tissue compared to BM-MSCs and ASCs grow faster that BM-MSCs during culture expansion and adipose tissue can be acquired by a simple minor liposuction in local anaesthesia[26–28] . Adipose-derived stem cells (ASCs) belong to the group of MSCs and are fibroblast-like and spindle-shaped adult stem cells. Adipose tissue naturally contains a high concentration of MSCs up to 30.000 ASCs/mL compared to other adult tissues such as bone marrow [29].

In recent years, there has been an increasing awareness that ASCs also have a number of striking paracrine bystander characteristics in terms of anti-inflammatory, anti-apoptotic, anti-fibrotic, immunomodulatory, angiogenic and trophic (tissue-regenerating) properties. In addition, it is has been shown that MSCs even by systemic infusion can identify areas of inflammation and tissue damage via a chemotactic gradient in the bloodstream. This chemotactic gradient (homing) causes the cells to adhere to the capillaries and post-capillary venules and from there migrate to the trans-lumen of the diseased tissue. The current hypothesis that has been thoroughly established is: MSCs act by secretion of paracrine factors in a "hit-and-run" scenario [30]. Recently, the theory has broadened to include secretion not only of cytokines and other soluble factors, but also extracellular vesicles that can contain cargos that include peptides, proteins, metabolites, microRNAs and even mitochondria [31].

The possible mechanism of action of MSCs is most likely not through engraftment but a supportive and paracrine function exhibiting anti-apoptosis, immunomodulation, angiogenesis, anti-scarring and support of growth, and differentiation of stem and progenitor cells [32].

The technique for the use of ASCs involves a mini- liposuction from either the patient (autologous cells use of ASCs) or a healthy donor (allogeneic use of ASCs) and isolation of the cell population of the stromal vascular fraction followed by in vitro expansion of the ASCs, which are in turn injected into the patient (autologous ASCs) or, as in our present study, the recipient (allogeneic ASCs).

### 1.3.2 Preclinical Studies: Mesenchymal stem cells in the treatment of xerostomia

For more than ten years, researchers have investigated the potential of mesenchymal stem cells as an approach to restoring the function of salivary glands after radiotherapy damage and several groups have addressed the use of MSCs/ASCs in preclinical studies of radiations-induced salivary

gland dysfunction. In 2013 our study group published a systematic review of the literature on ASC treatments for xerostomia: *Jensen DH et al.: Mesenchymal stem cell therapy for salivary gland dysfunction and xerostomia: a systematic review of preclinical studies. Oral Surgery, Oral Medicine, Oral Pathology and Oral Radiology 2013*[9]*.*

Today the leading belief about the beneficial action relies on paracrine pro-survival/proliferative, regenerative and immune-suppressive effects on the remaining epithelial stem cells and surrounding environmental cells. A proposed differentiation of bone marrow-derived MSCs has been observed in vitro; however, the actual contribution to epithelial differentiation in vivo is not clear but disputable[33]. A study with bone marrow-derived stem cells improved saliva function by epithelial repair and increased micro vessel density leading to improved blood perfusion of the glands. Evidence also indicates that ASCs diminish acinar cell apoptosis and reduce fibrosis [34,35]. Our group is currently conducting a systemic review to examine the newest preclinical and clinical evidence on safety and efficacy of mesenchymal stem cell therapy for radiation-induced salivary gland hypofunction and xerostomia.

### 1.3.3 Clinical Studies and our experience with mesenchymal stem cells for xerostomia

Only two papers are published with an implementation of ASCs into patients with postradiation xerostomia: one case report and our pilot study. The case report from 2017 is by Comella and Bell from the Stem cell Clinic in Florida and the South African Stem Cell Institute[36] . They describe a case with a 57 years old patient with severe xerostomia due to radiotherapy for at throat cancer, who received eight repeated ultrasound-guided transplantation of autologous ASCs into both submandibular and parotids glands. The patient received the first injection with 1.5 mL of stromal vascular fraction (SVF) plus platelet-rich plasma (PRP) containing approximately 30-60 x $10^6$ *ASCs* into each submandibular and both parotid glands on the day of the minor-lipoaspiration followed by seven injections of 2.5x $10^6$ culture-expanded ASC plus PRP in each submandibular and parotid glands at 5,8,16,18,23,28 and 31 month post- liposuction.

The patient was monitored 31 months after the liposuction, and no severe adverse events were reported. The patient demonstrated an increase in gland size measured by ultrasound, which corresponded to the self-reported increase in saliva production and increased quality of life.

Our research group has a substantial foundation and experience in conducting a clinical trial assessing mesenchymal stem cells as a possible treatment for the radiation-induced dry mouth. We have conducted to our knowledge, the first- in- man randomized, placebo-controlled, double-blind trial exploring the safety, feasibility and therapeutic effect of autologous adipose-derived stem cells as a treatment for radiotherapy-induced Xerostomia[11]. The study included 30 patients, randomized 1:1 ratio to receive an ultrasound-guided transplantation of ASCs or placebo to the submandibular glands. All participants had previously undergone radiotherapy for a T1-T2, N0-2A, human papillomavirus-positive oropharyngeal squamous cell carcinoma (OPSCC). The primary endpoint was the change in unstimulated whole salivary flow rate assessed by sialometry. All assessments were performed one month prior (baseline), and one and four months following the intervention of ASCs or placebo. In the study, no participants experienced severe adverse events towards the local injection of stem cells. Unstimulated whole salivary flow rates significantly increased in the ASC-arm at one (33%, P=0.0048) and four months (50%; P=0.008) after four

months, but not in the placebo-arm (P=0.6 and P=0.8) compared to baseline. In patient-reported outcome measurements (PROMs) the ASC-arm symptom scores significantly decreased on the xerostomia and VAS questionnaires, in the domain of thirst (-22%, P=0.035) after four months compared to baseline. The ASC-arm also showed significantly improved salivary gland functions of inorganic element secretion and absorption, at baseline and four months, compared to the placebo-arm. Core-needle biopsies showed an increase in serous gland tissue and a decrease in adipose and connective tissues in the ASC-arm compared to the placebo-arm (P=0.004 and P=0.02). Magnetic resonance imaging (MRI) showed no significant differences between groups in gland size or intensity.
Our group is at the moment gathering the results from the 1-year follow-up of the pilot study.

For an embracive description of our research group's previous work, experience, and results with mesenchymal stem cells for xerostomia, please sees our manuscripts for the protocol of MESRIX-I and the accepted publication of the results from the pilot study:
*Christian Grønhøj et al.: First-in-man mesenchymal stem cells for radiation-induced xerostomia (MESRIX): study protocol for a randomized controlled trial, Trials 2017*[10] and
*Christian Grønhøj et al.:* Safety and Efficacy of Mesenchymal Stem Cells for Radiation-Induced Xerostomia: A Randomized, Placebo-Controlled Phase I/II Trial (MESRIX), International Journal of Radiation Oncology, Biology, Physics, April 2018[11].

## 1.4 Allogeneic Stem Cells

### 1.4.1 Rationale for shifting from autologous to allogeneic ASCs

The aim of using allogeneic ASCs from healthy donors is to minimize and pre-emptive the variability in the investigational medical product (the ASCs) and pursue an ASC-therapy that is possible to integrate into an everyday clinical setting and avoiding liposuction.
The change in the origin of the stem cells is necessary to overcome the impediments with autologous stem cells. When using autologous cells, the complicated culture expansion takes weeks with a substantial individual yield in cell and growth and every patient must undergo liposuction. Avoiding liposuction is both a benefit for the slender former cancer patients, for the burden of the surgical department and for an economic perspective.
Both preclinical and clinical and clinical studies have shown that patient demographics such as age, comorbidities and prior treatment with cisplatin-chemotherapy affect the regenerative potentials and the fitness of MSCs[37–40].
The head and neck cancer patients are mostly above fifty years, often have comorbidities and cisplatin is a concurrent treatment for OPSCC stage III-IV according to American Society for Radiation Oncology (ASCO) 2017 guideline[41]. Therefore, the patient population likely to need mesenchymal stem cells therapy for restoring salivary function may not have ideal mesenchymal stem cells for ex-vivo expansion (for autologous therapy). In addition, most of the patients suffering from salivary gland hypofunction and xerostomia after radiotherapy have difficulties eating, which results in a very slender figure, which are not ideal candidates for liposuction.

Production of autologous ASCs was a vast hurdle in our pilot study as 2 of 18 participants (randomised to receive autologous stem cells) failed to complete the trial due to an inadequate number of mesenchymal cells expanded ex-vivo. Thus, more than 10% randomised to ASCs were excluded due to an inadequate number of MSCs and never received the intervention. Further, there was considerable variation between patients in cell yield and expansion time, which made the production of autologous cells immensely complicated and expensive.

Hence, a source of highly regenerative disease-free donor stem cells from young healthy persons, whom has no cancer history nor received chemotherapy, would dramatically advance the clinical translation of ASC treatment to patients with possible multiple comorbidities and a more cost-effective treatment with allogeneic compared to autologous.

### 1.4.2 Safety of allogeneic mesenchymal stem cells

Allogeneic MSCs were first used clinically more than a decade ago to treat severe steroid-resistant acute graft-versus-host disease (aGVHD), which demonstrated the capacity of MSCs to modulate allogeneic immune responses and treat human disease[42]. These finding lead to Canadas approval in 2012 as the first country in the world to allow a human allogeneic bone marrow mesenchymal stem cell therapy from healthy donors, *remestemcel-L/Prochymal*, for steroid-refractory aGVHD in children and since eight countries have followed.
These finding of MSCs disease fighting capacities made research in mesenchymal stem cells soared and MSCs/ASCs have since been intensively investigated in vitro, in laboratory animal experiments and at various evidence levels for multiple diseases. Multiple robust human trials in a variety of diseases have been conducted with allogeneic MSCs and ASCs without serious or systemic side effects and have shown promising clinically results [43–51]. Strong double-blinded, placebo-controlled randomised multicentre clinical trials (RCT) studies have demonstrated safety with limited local SAEs, and significant clinical results of one single local intralesional injection of allogeneic MSCs and ASCs for complex perianal fistulas in Crohn Disease (CD) including a maintained of safety and efficacy after 52 weeks [44,51,52]. The evidence gathered on the safety and efficacy in perianal fistulas in CD lead to the first EU approval in March 2018 of an "off-the-shelf" allogeneic adipose-derived mesenchymal cell therapy product, *arvadstrocel*.

Substantial evidence has been gathered of the use of allogeneic MSCs and ASCs for cardiological conditions with favourable results and safety in both intravenous and transendocardial stem cell injections (TESI)43,48–50,53. Results from the cardiological trials indicate that mesenchymal stem cells are safe to inject directly into the myocardium and the stem cells regenerate local tissue injury in the heart. CSCC_ASC has already proven safe in a phase 1 clinical trial for ischemic heart disease 50. A follow-up five years after a multicentre study treatment with intravenous transplantation of allogeneic MSCs without donor-matching for drug-resistant Systemic lupus erythematosus (SLE) illustrated both long-term safety and efficacy in clinical remission of the disease and improvement in organ dysfunction[47,54]. The same group have investigated the efficacy

of single versus double transplantations of allogeneic mesenchymal with no enhancement of efficacy of two stem cells transplantations compared to one in refractory SLE patients[55].

### 1.4.3 Efficacy of allogeneic mesenchymal stem cells versus autologous

No consensus exists about the best cell type or source for clinical regenerative therapy[26]. The presence of MSC-like cells in most tissues of the body is believed to indicate their importance, and preclinical studies indicate that these cells have regenerative capacity regardless of tissue origin [26,56].No definitive clinical advantage of autologous MSCs over allogeneic MSCs has been demonstrated to date and long-term studies have been published with follow-up safety and efficacy up to 8 years after treatment with allogeneic MSCs[30,47].

In 2017 Hare et al. published the results from the POSEIDON-DCM trial with randomised comparison of the safety, efficacy, and immune of allogeneic versus autologous mesenchymal stem cells for non-ischemic dilated Cardiomyopathy (NIDCM) with patients randomised 1:1 to receive 100 x $10^6$ ASCs trans-endocardial injection directly in the left ventricular. Results from POSEIDON-trial demonstrated treatment with both autologous and allogeneic MSCs were safe with no serious treatment-emergent AEs within 30-days and no differentiation in the incidence of AEs between the two treatment cell sources. The long-term safety follow-up at 12 months post - intervention found lower SAEs in the group receiving allogeneic MSCs compared to the autologous MCSs and significantly lower all-cause re-hospitalization rate in the allogeneic-MSC group. Interestingly, they found a greater efficacy of allogeneic MSCs over autologous MSC. Their study indicates no change in efficacy with allogeneic stem cells, which could have been suspected due to a possibly higher risk of immunological clearance.

As research in allogeneic mesenchymal stem cells therapy has soared the last ten years, the need for knowledge of the implications of immune response in the recipients is becoming ever more crucial. Culture expanded MSCs express low levels of HLA class I major histocompatibility complex (MHC) and lack expression of MHC class II surface molecules, and co-stimulatory molecules (e.g., B7-1, B7-2, CD 30, CD40 and CD 86), the MSCs have been rendered hypoimmunogenic[30] . This have led to a prevailing dogma that allogeneic mesenchymal stem cells are immune privileged but only a very scarce number trials control for matched or mismatched major histocompatibility complex (MHC) molecule expression examine the immunogenetic in vivo[57]. Recent clinical studies and reviews of development of donor-specific antibodies (DSA), suggest that allogeneic MSC/ASCs are not immune privileged rather immune evasive[30,44,50,58]. In multicentre study by Panes et al. the immune response was investigated and they found 16 % patients in the treatment group and 15% in the placebo group had pre-existing donor-specific antibodies (DSA, IgG HLA class I antibodies) at baseline [44]After 12 weeks 34% of the ASCs treated patients and none of the placebo-treated patients, who were tested negative at baseline generated donor-specific antibodies. They did not find association between positivity for donor-specific antibodies and therapeutic response.

A recent safety study by Kastrup et al. with transplantation of allogeneic ASCs directly in the myocardium did not cause serious adverse events[50]. They found that two out of ten already had donor-specific HLA antibodies (DSA) at baseline and four out of 10 patients developed de novo donor specific HLA class I antibodies but no changes in inflammatory parameters and the development of DSAs no did not influence the efficacy of the ASCs. Influence of HLA-matching on the efficacy of allogeneic MSCs was assessed in two clinical trials with osteoarthritis and degenerative disc disease. The studies demonstrated only weak and transient immune response with reactivity decaying during the first year and not enhancement of efficacy with donor-recipient HLA matching [58].

In animal studies the majority of allogeneic stem cells die within 48h after systemic infusion[30,59]. The assumption of early MSC death after infusion and no engraftment of allogeneic ASCs were recently confirmed through analysis of tissues at autopsy of 18 patients who received MHC-mismatched allogeneic MSCs. No ectopic tissue was observed and only one severely immunocompromised patient showed high levels (>1/1,000 cells) of donor DNA in multiple tissues [30,59]. The current evidence indicates that allogeneic MSCs/ASCs are safe however solid randomised clinical trials assessing the immune response within the recipients are warranted, which we aim to conduct if the current study find the treatment safe to transplant into the salivary glands.

## 2. RESEARCH HYPOTHESIS

Treatment with allogeneic adipose-derived mesenchymal stem cells will result in an improvement of the participant's unstimulated and stimulated whole saliva flow rate and ameliorate the xerostomia and increase quality of life.

## 3. STUDY OBJECTIVES AND ENDPOINTS

### 3.1 Study Objectives

The study objectives are to:

- to evaluate the safety and tolerability of treatment with allogeneic adipose tissue-derived mesenchymal stromal/stem cells CSCC_ASC(50) transplanted into both submandibular and parotid glands in patients with radiation-induced hyposalivation and xerostomia.
- to evaluate the feasibility and efficacy of the treatment with allogeneic adipose tissue-derived mesenchymal stromal/stem cells CSCC_ASC(50) transplanted into both submandibular and parotid glands assessed by change in salivary flow after one and four months compared to baseline in patients with radiation-induced hyposalivation and xerostomia.

### 3.2 Study Endpoints

#### 3.2.1 Safety Endpoints

1. Safety evaluated by the development of SAEs and SUSARs in four months (primary endpoint).
2. Immune response to allogeneic ASC. Measured by development of de novo human leucocyte antigen antibodies (HLA) and a diversity of immune mediated cytokines (an array of immune-related markers will be analysed) and Truculture.

#### 3.2.2 Efficacy Endpoints

1. Change in salivary gland function measured by a four month change in unstimulated whole saliva flow rate.
2. Change in quality of life. Evaluated by a change in complaints of xerostomia evaluated by two patient questionnaires (QLQ-H&N-35 and XQ).
3. Change in saliva gland function assessed by salivary gland 99mTc scintigraphy after 4 months.
4. Change in the composition of saliva after one and four months, respectively

#### 3.2.3 Exploratory Endpoints

1. Correlation of efficacy and immune response

# 4. INVESTIGONAL PLAN

## 4.1 Overall Design and Plan of the study

The study is an investigator-initiated, prospective, single-Centre, open-label trial to investigate the safety, feasibility and efficacy of allogeneic adipose tissue-derived mesenchymal stem cells (ASCs) as a treatment for radiation-induced hyposalivation and xerostomia for ten previous oropharyngeal cancer patients. The population will represent 10 patients with severe complications and reduced quality of life after radiotherapy. The study will evaluate the safety and efficacy of allogeneic adipose- tissue-derived mesenchymal stem cells with using CSCC_ASC (50). Because the allogeneic ASC product has not been tested in human salivary glands, this study is designed as an open-label Phase I study.

The first 5 patients will receive the IMP with 5% DMSO and the last 5 patients will receive the IMP with DMSO 10%. The first two participants in each DMSO-group (patient 1-2 and patient 6-7) will at the intervention (baseline) have the IMP transplanted into both submandibular glands and the right parotid gland, come again after 24 hours for checkup and blood samples. After seven days the first two patients will return for checkup and have the IMP injected into the left parotid gland.

### 4.1.1 Step-by-step description of the study

- *RECRUITMENT OF PARTICIPANTS*: Recruitment of participants with hyposalivation and xerostomia after radiotherapy for an oropharyngeal cancer. Information and informed consent.

- *SCREENING TESTS*: Maximum 60 days prior to intervention
  - The first step after informed consent for possible study participants is to have their usual medical history taken according to guidelines from the Ear, Nose and Throat Surgical Department, Copenhagen University Hospital Rigshospitalet. The possible participants will have blood samples analysed for anti- HIV- I and II, syphilis, anti-HBsAg, anti- HBc and anti-HCV, as well as relevant kidney parameters, and be tested to evaluate renal function. The tissue and blood type will also be evaluated (if unknown). The participant subsequently undergoes saliva flow measurements, fills out quality of life questionnaires and has an ultrasound exam of the salivary glands.
  - The participants will have a salivary gland scintigraphy performed.
- *BASELINE: INTERVENTION*:
  - On the day for the intervention the participants will have blood samples taken to evaluate inflammatory activity and the presence of Human Leucocyte antigen

antibodies (HLA antibodies). The tissue and blood type will also be evaluated (if unknown).
    - The intervention with CSCC_ASC(50) will be ultrasound-guided injected to the parotid and submandibular glands.
- *FOLLOW-UP: 1 DAY AFTER INTERVENTION (Maximally 2 days after intervention)*
    - The participants will perform a sialometry and have blood samples

- FOLLOW-UP: 5-7 DAY AFTER INTERVENTION
    - Intervention of the contralateral parotid (only patient 1-2 and 6-7)
    - The participants will perform a sialometry and have blood samples (all patients 1-10)

- *FOLLOW-UP: 1 MONTH AFTER INTERVENTION (+/- 7 days)*
    - The patients will undergo saliva flow measurements, have blood samples taken and fill out quality of life questionnaires
- *FOLLOW-UP: 4 MONTHS AFTER INTERVENTION – LAST VISIT (+/- 45 days)*
    - The patients will undergo the final saliva flow measurements and fill out quality of life questionnaires
    - The participants will have salivary gland scintigraphy performed
- *YEARLY SAFETY AND EFFICACY FOLLOW-UP* (schedule of tests attached as appendix II)
    - The patients will be contacted by phone and invited for a check-up
    - The patients will be monitored for five years.

# 5. STUDY POPULATION

## 5.1 Justification of study population and number of participants

10 former oropharyngeal HPV positive or HPV negative patients with radiation-induced hyposalivation and xerostomia are planned to be enrolled. As in MESRIX-I, we will standardize the participant population by excluding patients with severe salivary gland hypofunction and already manifest or near-manifest xerostomia, as this participant population most likely will not benefit from the treatment. After informed consent, the patient's symptoms and salivary gland function will be screened by a preliminary questionnaire and a subsequent saliva flow rate measurement before inclusion.

## 5.2 Inclusion criteria

1. Age between 18-75 years
2. Previous radiotherapy +/- chemotherapy for OPSCC stage I- II (UICC-8, 2017).
3. 2 years' follow-up without recurrence
4. Clinically reduced salivation and hyposalivation, evaluated by a screening
5. Unstimulated salivary flow rate between 0.2mL/min and 0.05mL/min
6. Grade 1-3 xerostomia as evaluated by the UKU side effect rating scale
7. WHO Performance status (PS) 0-1[2]
8. Informed consent

## 5.3 Exclusion criteria

1. Any cancer in the previous 4 years (not including OPSCC and basocellular carcinomas)
2. Xerogenic medications
3. Penicillin or Streptomycin allergy
4. Any other diseases of the salivary glands, e.g. Sjögren's syndrome or sialolithiasis
5. Previous parotid or submandibular gland surgery
6. Previous treatment with any type of stem cells
7. Pregnancy or planned pregnancy within the next 2 years
8. Breastfeeding
9. Smoking within the previous 6 months.
10. Alcohol abuse (consumption must not exceed 7 units/week for women and 14 units/week for men (Danish National board health alcohol guidelines[3])
11. Any other disease/condition judged by the investigator to be grounds for exclusion

## 5.4 Criteria for withdrawal from the study

Participants can withdraw from the study at any time without reason. Should a participant decide to withdraw before the intervention they *screen failure*. Data from patients that withdraw from the study after the intervention will still be included in the data analysis. For drop-outs all efforts will be made to complete and report the observations. All participants, who have received the MSCs treatment will be asked to roll-over to the long-term follow-up (LTFU) protocol upon premature discontinuation of any reason including withdrawal of consent.

The sponsor Christian von Buchwald and the principal investigator Charlotte Lynggaard can exclude/withdraw participants for safety reasons at any time during the study period

### 5.4.1 Criteria for withdrawal from the study before treatment

1. Pregnancy Infection of the transplanted site
2. Withdrawal of consent from participant
3. Cigarette smoking
4. Cancer (recurrence or new primary cancer)

## 5.5 Contraception

All women of childbearing potential attending the study are obligated to use highly effective contraceptives to according to the CTFG2014 recommendations to avoid pregnancy during the four months study period.  Women are considered of childbearing potential, i.e., fertile, following menarche and until becoming post-menopausal (> 12 months of amenorrhea in senior women) unless permanently sterile. Permanent sterilization methods include hysterectomy, bilateral salpingectomy, and bilateral oophorectomy. Highly effective contraceptives are defined as hormone pills (combined hormonal contraception associated with inhibition of ovulation or progestogen-only hormonal contraception associated with inhibition of ovulation), bilateral tubal occlusion, an intra-uterine device (IUD), intrauterine hormone-releasing system (IUS) or vasectomized partner (provided only one partner).  Contraceptives are not required if the participants are not female. To enter the study younger female will have excluded pregnancy by HCG.

# 6. METHODS

## 6.1 The dose of the study drug

In our pilot study, the participants solely had stem cells transplanted into the submandibular glands. The participants in MESRIX-I received a cell dose of 2.8 x $10^6$ ASC/$cm^3$ gland, i.e., a maximum of the total number of approximately 50-100 x $10^6$ ASCs per participant without side effects. The size of the submandibular glands relied on both MRI-scans and ultrasound. The number of ASCs used in MESRIX-I was based on published animal studies in which the amount given for xerostomia to mice varied from 2 x $10^5$ to 2 x $10^6$ [9]. In this present study the participants in addition will have stem cells transplanted into the parotid glands. The size of the submandibular gland in human subjects after radiotherapy is 6.6 $cm^3$, and the volume of the parotid gland after radiation is approximately 15.73 $cm^3$ We found that the size of the submandibular glands variated in size from 2 $cm^3$ to 21 $cm^3$ [60]. In this present study the participants in addition will have stem cells transplanted into the parotid glands. With ultrasound it is possible to visualize and assess the size of the submandibular glands, and the superficial lobe of the parotid gland. The deep lope of the parotid is obscured by the acoustic shadow of the mandibular ramus[61]. Therefor it is im-possible with ultrasound to measure the size of the parotid gland. As is not feasible in a clinical setting for all possible stem cells candidates to undergo MRI, the parotid glands will receive a fixed dose. The size of the submandibular glands will rely on the size estimates by ultrasound combined with the measures from salivary gland scintigraphy. This setup will be easier to convert for possible future implementation of the ASC therapy into clinical practice. Based on our experience from our previous study, we increase the numbers of ASC and test a standard dose with 50x$10^6$ for parotid glands and 25 x$10^6$ for submandibular glands < 10 $cm^3$ and 50x$10^{6\ for}$ submandibular glands above 10 $cm^3$. Participants will receive approximately a dose of 150-200x $10^6$ when treating the four major salivary glands.

## 6.3 Technique for intraglandular administration of ASCs into the salivary glands

Subjects will go to the ENT Department at Rigshospitalet, section 2073 for outpatient procedures. Here it will be evaluated whether they are ready for injections. After receiving the suspension of stem cells, the surgeon will together with a second authorized health staff witness the identification of the patient and suspension number. Hereafter the surgeon will identify submandibular and parotid glands, and ultrasound-guided inject the suspension of ASCs with the corresponding identification number. The suspension of ASCs will be deposited in 2 areas in each gland to secure a safe and equal distribution of the suspension. This procedure is the same as in our previous study and was chosen to ensure a safe and homogeneous distribution of the suspension in the parenchyma[62]. In our pilot study, there were no reports of adverse events due to this procedure[11] . Afterwards, the participant will be given a Band-Aid to be removed the following day and over the counter analgesics.

# 7. STUDY ASSESSMENTS

## 7.1 Assessments of safety

### 7.1.1 Evaluation, reporting and recording of adverse events

All adverse events (AEs) are monitored and recorded along with concomitant medicine at the scheduled follow-up (day one, 1 month and 4 months after the intervention). AEs are defined as any untoward medical occurrence in the clinical trial participant administered a medicinal product and which does not necessarily have a causal relationship with the treatment (ASCs). AEs will be assessed and graded according to Common Terminology Criteria for Adverse Events v5.0 guidelines (CTCAEv5.0). Thus, all adverse events are recorded with CTCAE grade. All grade 3 and grade 4 events and incidents considered related to this trial will be reported to the sponsor by the investigator immediately after they are discovered.

An adverse reaction (AR) is defined as any untoward or unintended response in a participant to an investigational medical product (IMP) which is related to any dose administered to that subject. An adverse Drug Reaction (ADR) is stated as all noxious and unintended responses to a medicinal product related to any dose should be considered adverse drug reactions.
Serious Adverse Event (SAE), Serious Adverse Reaction (SAR) or Suspected Unexpected Serious Adverse Reaction (SUSAR), means any AE, AE, SUSAR, respectively, that:

1. Results in death or
2. A serious deterioration in health that
    a. Resulted in life-threatening illness or injury
    b. Required hospitalisation or prolongation of existing hospitalization
    c. Resulted in permanent impairment of body structure or body function
    d. Resulted in medical or surgical treatment to prevent the above
3. Lead to foetal death, congenital anomaly or birth defect, or other negative effect on the foetus
4. Anything the Principal Investigator deems to be of Clinical serious significance

Medical judgement will be exercised by the primary investigator together with sponsor whether an adverse event/reaction should be classifies as serious in other situations.
The included participants will be instructed to contact the principal investigator or a special trained person from the research group in case of events with possible relation to the trial treatment within the main study period.
The principal investigator will immediately (within maximum 24 hours after receiving the information) inform the sponsor if serious or serious suspected adverse reactions occur. In our previous study MESRIX-I with autologous ASCs no SAR or SUSARs were detected, so any SAR in this study will be reported as a SUSAR. The sponsor will report SUSARs (Suspected Unexpected Serious Adverse Reaction) to the Danish Medicines Agency and the Danish National Committee on Health

Research Ethics within 15 days of occurrence and 7 days in case of death or life-threatening issue. Unexpected reactions are defined as reactions which are not described in the section "Reference Safety information" of this protocol.
All other unexpected serious adverse reactions will be reported within 15 days after the sponsor becomes aware of these.
All serious adverse reactions (SARs) that have occurred during the trial will be reported to The Danish Medicines Agency, The Danish Patient Safety Authority, and the National Committee on Health Research Ethics (if appropriate) once a year by the sponsor.
The sponsor will annually send a concise review and evaluation of pertinent safety information, a Development Safety update report (DSUR) collected during the reporting period related to the Danish Medicines Agency, DKMA.
After completion of the trial, the sponsor will after 90 days inform the Danish Medicines Agency that the study is completed. Within a year, the sponsor will submit trial results to the board, according to the law on drugs (Lov om lægemidler) § 89. 2, No. 4

### 7.1.2 **Table 1:** Adverse events with possible relation to injection of ASCs and FNA

| | | |
|---|---|---|
| **Type** | **Frequency** | **Seriousness** |
| Temporary soreness of the salivary gland | 10-50% | Not serious |
| Temporary redness of the salivary gland | 10-50% | Not serious |
| Temporary swelling of the salivary gland | 10-50% | Not serious |
| **Adverse events with relation to similar procedures and in MESRIX-I** | | |
| **Type** | **Frequency** | **Seriousness** |
| Temporary soreness of the salivary gland (up to 7 days after injection) | 10-20% | Not serious |
| Temporary redness of the salivary gland (up to 7 days after injection) | 10-20%% | Not serious |
| Temporary swelling of the salivary gland (up to 7 days after injection) | 10-20% | Not serious |
| Infection of the salivary gland | 0.5-5% | Not serious |
| Bleeding/hematoma in the salivary gland | 1-5% | Not serious |

The above numbers and adverse events are based on our previous phase I-II trial (**EudraCT: 2014-004349-29**) and on the literature cited and described in the section "*Risk Assessment*".

In our previous study MESRIX-I with autologous ASCs no SAR or SUSARs were detected, so any SAR in this study will be reported as a SUSAR.

## 7.2 Long-term safety and efficacy follow-up

Following CHMP under EMA draft from January 2018 on safety and efficacy (S&E) follow-up and risk management of advanced therapy medicinal products (ATMP), the participants will be invited to a follow-up of efficacy and adverse reactions approximately every second year for five years after the intervention. The long-term follow-up (LTFU) will include basic ENT-examination, fiberscope, ultrasound of salivary glands, sialometry, scintigraphy and blood samples to follow immune response. Data from the electronic medical journals and national pathology database will be gathered. It is expected that a number of the participants will have a recurrence of their primary cancer during the study or follow-up period due to the nature of the OPSCC. The allogeneic mesenchymal stem cells are not expected to engraft in the salivary glands the recipients as the stem cells are adult non-pluripotent. However, in case of recurrence of cancer or new primary cancer developments each case will be carefully evaluated and *potentially* investigated with genetic analysis to distinguish whether a tumour is due to recurrence, the administered product or endogenous tumour formation.

## 7.3 Assessments of efficacy

### 7.3.1 Sialometry

There is no accepted golden standard for diagnosing salivary hypofunction or evaluating the function and flow rates of the major salivary glands, however sialometry is the most advocated method to evaluate the unstimulated and stimulated whole saliva production. Sialometry is a direct measurement of the flow rate in either unstimulated or stimulated state of secretion. This test is valuable since it is easily quantifiable [63].

A change in the secretion rate of the unstimulated whole saliva in the oral cavity is probably the most deciding parameter for the biological development of hyposalivation and xerostomia and accompanying pathological oral conditions. Whole saliva is the secretions from the major and minor salivary glands, which are mixed in the oral cavity. A correct determination of this value is crucial for the assessment of treatment outcome in this project.

For assessment of the saliva flow rate, whole saliva will be collected between 9 a.m. and 12 a.m. or 1 p.m. and 4 p.m. (each participant will have *all* the saliva tests done either morning or afternoon). The participants need to drink at least 2 L of water the day before a sialometry. Subjects will be prohibited from eating, drinking, and performing oral hygiene for 1 hour before the collection. After being seated upright in a chair, they relax for 5 minutes and are then instructed to make as few movements as possible, including swallowing, during the collection. Before and after treatment, unstimulated whole saliva will be collected using the spitting method in which participants spit their saliva into a collection container over a period of 10 minutes[64]. The

salivary flow rate (SFR) (mL/min) is determined by the increase in weight of the container divided by the collection time in minutes.
After the collection of unstimulated saliva, the subjects are instructed to chew on 1g of sterile paraffin wax. They will be asked to keep their mouths closed during chewing and to avoid swallowing. Every 60 sec they will be asked to spit into a new saliva collector before starting the next chewing period. This will be repeated for 5 min.

### 7.3.2 Salivary Gland Scintigraphy

Salivary gland scintigraphy has been used for decades to assess the function of the major salivary gland function including post-radiation dysfunction[61]. Salivary gland scintigraphy is minimally invasive, has high patient tolerance and compliance, has a low radiation dose, and has no interference with the normal physiology of the salivary glands[65]. The assessment provides results for several parameters of the salivary gland function by the use of the absorption and excretion properties of radioisotopes such as $^{99m}$Tc. [66]. The $^{99m}$Tc. is readily trapped and secreted in the ductal epithelium of salivary glands and excreted in the saliva, which allows salivary gland scintigraphy to be performed and also provides quantitative information on the glandular function[65].

### 7.3.3 Assessment of the quality of the saliva: Analysis of saliva

Whole saliva contains a large number of bacteria and epithelial cells as well as gingival crevicular fluid. Therefore, whole saliva is usually not suited for the analysis of sensitive chemical parameters. As in our pilot study, we will analyze the following on the collected saliva: pH and bicarbonate by ionic balance estimation[67], sodium, potassium, calcium, phosphate, chloride and fluoride[68], total protein and selected proteins and[69] amylase[70,]. The purpose of these studies is to evaluate whether saliva will be normalized after treatment, and thus provide an estimate of the saliva dental and mucosal protective capacity before and after the intervention, and to analyze if we can measure any activation. Furthermore, in the present study saliva samples will be analysed for a panel of cytokines using ELISA assays. Cytokines in saliva are known to participate in the inflammatory and immune responses, and we aim to detect inflammatory activation due to the injection of ASCs[71]. Further, we will analyze changes in mRNA and proteomics in the saliva. For optimal value of the saliva analyses we request the participant to undergo a Dental examination prior to participating in the study or to share a copy of their last dental examination **and to have panoramic radiography (dental x-ray) performed.** For optimal interpretation of the saliva samples we ask 10 healthy age-matched control, who have not undergone radiotherapy to donate a saliva sample after standardized sialometry. **The control group like the participants will have panoramic radiography (dental x-ray) performed and undergo dental examination and/or share a copy of their dental journal.** The control group will be recruited by asking, if relatives of the particip**ants, relatives to former OPSCC patients, patients and relatives to patients and colleagues** will donate a saliva sample.

### 7.4 Patient-Reported Outcome: Methods for evaluating xerostomia

To evaluate the participants´ perception of xerostomia, the participants will answer validated questionnaires in Danish (EORTC QLQ Module for H&N-35 and QX) at baseline, after one month and again after four months. These patient-reported outcomes (PROMs) questionnaires are essential, validated tools for estimating the degree of quality of life (QoL) and xerostomia[72,63]. In our predecessor study, MESRIX-I, the patients self-reported their symptoms with the Xerostomia Questionnaire (XQ). In this present study, participants will have a chance to evaluate more overall implications of the xerostomia with the Quality of Life Head and Neck-35 questionnaire, which also examines possible social and sexual problems secondary to xerostomia.

## 8. DATA ANALYSES

This is an observational Phase I safety study. All adverse events will be reported. Functional changes in saliva production will be measured by a four month change in unstimulated whole saliva flow rate.

## 9. BIOBANKS RELATED TO THE STUDY

Two biobanks are necessary for the study, all under the responsibility of Charlotte D. Lynggaard and Christian von Buchwald:

1. Salivary fluid
2. Blood samples from the participants

## 10. ETHICAL CONSIDERATIONS

The trial is conducted in accordance with the Helsinki II declaration. The National Committee on Health Research Ethics will be requested permission to conduct the study. Further will request the Danish Data Protection Agency for assistance to guarantee the trial follows the newest version of the General Data Protection Regulation. Moreover, the Danish Medicines Agency will be requested approval, and the trial will be GCP monitored (Birgitte Grøn). Each participant is required to give written informed consent before he/she can be included in the study. Information obtained about participants' health, other purely private matters and other confidential information is covered by professional secrecy. No project participant has a personal financial incentive to implement the described project. The samples will be analysed and stored in Denmark.

# 11. RISK ASSESSMENT AND BENEFITS

## 11.1 The procedure

Injections of ASCs into the submandibular and parotid salivary glands are associated with the risk of adverse events such as infection and bleeding is estimated to be below 0.5 % when using similar procedures. In our previous study, none of the 30 participants developed serious adverse reactions[73].
In the present study ASCs will also be transplanted into the parotid gland, where facial nerve resides. The participant may also experience some pain briefly during injection of the transplant product (ASCs).

## 11.2 Mesenchymal/adipose-derived mesenchymal stem cells

A theoretical risk of a possible carcinogenic effect in the treatment with MSC/ASCs has been discussed. This is due to their production of growth factors such as epidermal growth factor (EGF), a factor produced by the MSC/ASCs. These considerations are particularly relevant since the stem cells are used in participants previously diagnosed with cancer. However, in model systems of cancer, the effect of MSCs on cancer growth is controversial as they have both been shown to be inhibitory and stimulatory [74–78]. Further, numerous clinical trials with more than 1000 participants have been conducted with MSCs in different participant populations, and no increase in the incidence of cancer has been detected [79–81]. These studies include local injection of MSCs [82,83]. With respect to the use of ex vivo expanded MSCs, despite extensive research, there is no data indicating malignant transformation of expanded human MSCs/ASCs [79,84,85], including a study by Katz et al which demonstrated in a murine model that the injection of a very high dose of MSC ($240 \times 10^6$ MSC/kg) revealed no signs of cancer, organ toxicity or change in bodyweight after 12 months [86].

## 11.3 The study drug

The study drug allogeneic adipose-derived mesenchymal stem cells, CSCC_ASC(50), is an advanced therapy investigational medicinal product (ATIMP) manufactured from abdominal adipose tissue from healthy donors. CSCC_ASC(50) builds on the product CSCC_ASC presently in phase II clinical trials for ischemic heart disease, modified to meet treatment specific dosage needs for the indication radiation-induced hyposalivation and xerostomia in previous head and neck cancer patients. The product CSCC_ASC with DMSO 10% have safely injected directly into the myocardium in 135 patients.

CSCC_ASC(50) is manufactured according to GMP at Cardiology Stem Cell Centre, Rigshospitalet (aut no 23909), using manual isolation of cells from abdominal fat tissue, animal-free expansion in automated closed bioreactor systems and cryopreservation of the final product.

The active substance is aseptically prepared and in vitro expanded ASCs. The final product (CSCC_ASC(50)) is provided as a cryopreserved suspension of 55 million ASCs in 1,1 ml excipient. The product comes in two formulations for present clinical phase 1 safety testing: excipient holding 5% DMSO and 10% DMSO, respectively.

All healthy donors sign an informed consent complying with the declaration of Helsinki. Prior to donation donor eligibility is determined based on a donor interview, a questionnaire and testing for infectious disease markers. A donor is eligible only if the screening shows that the donor is healthy, and free from risk factors, and the laboratory tests for infectious disease agents are negative. Donor eligibility is determined and documented by two medical doctors independently. Each donor is tested for HIV, hepatitis B and C, syphilis and HTLV I/II serology by serum analysis within 30 days prior to liposuction. In addition, a blood sample is drawn on the day of donation for repeated serology and NAT (nucleic acid) testing of HIV, hepatitis B and C.
Liposuction is performed according to CSCC procedures and tissue license by a trained plastic surgeon and in full compliance with surgical procedures for sterile cosmetic surgery.

The CSCC_ASC(50) final product is tested sterile, mycoplasma- and endotoxin free. All biological raw materials used apply to European Pharmacopoeia (PhEur) chapter 5.2.12. Biological Raw Materials. Materials of human blood-derived origin are serology tested (infectious disease agents) and processed according to Blood Law, and pool sizes are considered according to PhEur directions. Recombinant proteins used come with certificates of origin (viral/TSE).

Final release of products is based on a GMP review of full batch documentation. Specifications are defined for intermediate ASC product batches, final ASC product batches and excipient used. ASC specifications include viral safety (donor serology), sterility (including mycoplasmas and endotoxins), cell number and viability of ASCs and immunophenotypical characterization of cells by flow cytometry. Excipient specifications include visual Inspection for particulates, pH, sterility (including endotoxins) and viability of cells following preservation.
Sterility, viability, immunophenotype and biological function of cells after 2 years of storage has been documented for CSCC_ASC. CSCC_ASC(50) stability studies have been initiated.
CSCC_ASC(50) is produced from multiple donors, but every batch/treatment unit is based on one donor only.
Further information about the quality of the study drug, including risks and benefits of CSCC_ASC (50) to human patients is found in the attached Investigational medical product dossier (IMPD).

CSCC_ASC (50) is composed of allogeneic ASCs cryopreserved using Cryostor (BioLifesolutions) with dimethyl sulfoxide (DSMO) as a cryoprotective agent (CPA). Currently cryopreservation is the only method to preserve cells for long-term[87]. Cryopreservation maintains cell functional properties and allows pooling of cells to reach the numbers required for clinical application[87]. Because cryopreservation bears a risk of cell injury, CPA is crucial to for survivability of the cells.

For clinical applications with MSCs/ASCs the most used CPA is DMSO 10%. The original CSCC_ASC product is made with 10% DMSO and has been injected directly into the myocardium in 135 patients without SAEs. As the treatment med CSCC_ASC(50) is the first time for an allogeneic ASC treatment of the major salivary gland in human the study is designed to start with 5% DMSO for the first five patient and increase to 10% DMSO in the last five patients. Further information on clinical experience with Cryostor is found the IMPD.

## 11.4 Scintigraphy

The patients will be exposed to a small radiation dose from the salivary gland scintigraphy of 250mBq$^{99m}$Tc per test. The total effective dose (0.012 mSv/mBq) of two tests is 6.5 mSv, which corresponds to two years of natural background radiation in Denmark. This increases the theoretical risk of cancer development from the usual 25% in the population to 25.003%.

## 11.5 Overall risk

The risk of cancer and risk of facial paralysis are assessed as minimal and acceptable. We have, however, taken the following safety measures to minimize risks in the study:

- Only participants with a previous disease stage of I-II
- Participants have been without recurrence for 2 years
- Participants are non-smokers, reducing the likelihood of field-cancerization
- The areas where the ASCs are injected to are not predisposed to cancer development
- The manufacturing process of ASC has been validated with the aim to maintain the number of population doublings at the minimum level needed to obtain the required number of cells without any effect on the genetic stability
- There is some distance from the injection site of the ASCs (salivary glands) to the place where the participants had their cancer (oropharynx).
- ASCs come from healthy donors without any cancer history
- The first two patients with DMSO 5% ( patient 1-2) and with DMSO 10% (patient 6-7) will have injected the IMP first in the right parotid gland and have clinical check-up after 24 hours and again after 7 days. If no sign of facial nerve paralysis the IMP will be transplanted into the contralateral left parotid gland.
- Participants will be monitored for 5 years after the intervention

Before the mini-liposuction harvest, the donors will have to undergo screening for microbiological agents (including syphilis, hepatitis-virus, and HIV), as is required by Danish national law. All participants will undergo testing for contagious infections (including syphilis, hepatitis-virus, and HIV), and should a participant sample unexpectedly be tested positive, the participant will be informed of the result, excluded from the study and referred to the Department of Infectious Diseases, Rigshospitalet, for follow-up and subsequent care.

## 11.6 Benefits of the participants

It is estimated that the overall risks of adverse events in the experiment are outweighed by the benefits of participation. Our previous study proved clinical safety and efficacy of intraglandular treatment of the submandibular gland with autologous ASCs with both increased production of saliva and improvement in patient-reported outcomes. Treatment with allogeneic ASCs is expected to give rise to similar benefits for the individual patient as did autologous ASCs. In the present study the all participants will receive treatment with allogeneic ASCs into both the parotid and submandibular glands. The submandibular gland is the most important for the production of unstimulated saliva, whereas the parotid gland accounts for around 65 % of the stimulated saliva production. It is an improvement to treat both of the largest saliva glands. The facial nerve is embedded between the superficial and deep lope of the parotid gland. The ASCs injection is made ultrasound-guided into the superficial of the parotid. We do not expect for the study drug or the injection to harm the facial nerve as the CCSC_ASC have been safely injection the myocardium, which has both sympathetic and parasympathetic nerve running within.

As an extra precaution, the study is designed to start with CSCC_ASC (50) with 5% DMSO for the first five patients. For the first two patients, they are treated on the right parotid gland and if no complication they will have the injection of ASCs into the contralateral parotid one week later.

For inclusion in the study, subjects will be informed that in the current study every participant will receive ASCs from healthy donors, which could theoretically lead to the transmission of known or unknown viruses, that the cells will be examined for a number of known viruses, and that the risk of viral transmission is considered minimal. The participants will also be informed of the risk that the participants will develop donor specific de novo human leukocyte antigen class I antibodies (HLA).

The development of a method for treating xerostomia which is efficient, safe and reproducible will benefit the participants and future head and neck cancer patients undergoing radiotherapy. If the treatment has a clinically significant effect, patients suffering from radiation-induced xerostomia in the future could be offered a simple, minimally invasive procedure with few adverse events and risks, which is in contrast with the current sub-optimal treatments.

## 12. DATA COLLECTION

Source data: There will be source documentation for all data in Case Report Form (CRF).
The study director allows direct access to study data and study documents for the monitoring, audit, and inspection of the Science Ethics Committee, the Danish Health and Medicines Authority or similar authorities in other countries. Permission will be sought from the DPA for the processing of personal data under the General Data Protection Regulation. Applications will be sent via the legal secretariat, Rigshospitalet. Data will be analyzed with R. Our data will be kept securely in the electronic data capture system (EDC) Redcap. All data will be directly entered. The trial will be completed when all data is collected. All results will be stored and analysed electronically; participants' anonymity is ensured in accordance with the national data legislation. After completion of the study, data will be stored in anonymous form. Data containing social security numbers will be kept locked and inaccessible to unauthorized persons.

# 13. RECRUITMENT OF PARTICIPANTS AND OBTAINING INFORMED CONSENT

## 13.1 Guidelines for submission of the oral information and obtaining consent

### 13.1.1 Recruitment of participants and obtaining informed consent

The study participants will be recruited through the ENT Department of Rigshospitalet and Oncology Departments at Rigshospitalet and Herlev Hospital. The investigator will send a letter to eligible patients who have been treated and followed for their previous cancer disease, and subsequently the principal investigator or a trained member of the study group will contact patients by phone and inquire about their interest in participating in the research project. If they have an interest in participating in the project, they will be invited to an interview at the outpatient clinic of the Department of Department of Otolaryngology, Head and Neck Surgery, Rigshospitalet for further information concerning the trial. In the written information material, besides material about the trial, the pamphlets" Forsøgspersoners rettigheder i et sundhedsvidenskabeligt forskningsprojekt"("Rights of test subjects in a health scientific research project") og "Før du beslutter dig" will be included. The right to bring counsel to the information interview will likewise be explained. Motivated patients referred from doctors in other parts of Denmark and patients who approach our department with an interest in participation in our stem cell research will also be offered information about the trial. Patients from all parts of Denmark can be included if they pass the inclusion and exclusion criteria described below. However, transportation and accommodation cannot be provided.
The information interview will take place in an undisturbed environment with the principal investigator or trained member of the study group, who has the professional qualifications to communicate the content of the research project, and who will clearly explain that it is a request to participate in a health science research project. The participant will be given the oral information about the project in layman's terms based on the written information, and any questions will be answered. The oral information will be adapted to the participant's requirements, and it will be explained without the use of technical terms. Participants will be informed of the right to reflection following the information interview. Participants will likewise be informed of the opportunity to get feedback on the scientific results. The potential participants for study will be contacted by telephone by the responsible physician for final commitment to participate. If participants are still interested in participating in the project, an appointment is made where a medical history is taken, and where the remaining trial appointments will be planned in agreement with the participant, and here the participants and the responsible physician will sign the medical consent form.

## 14. SUBJECT CONFIDENTIALLY

The investigator is obliged to ensure that participant anonymity is protected and maintained. On the CRF´s subjects should be identified by their initials and a subject study number only, Documents that are not for submission (e.g., signed informed consent forms) will be kept in strict confidence by the principal investigator. In compliance with the GCP Guidelines, it is required that the investigator and the institution permit authorized representatives of a monitoring company direct access to reviewing subjects' original medical records for verification of study related procedures and data. All information obtained concerning this protocol regarding participants is protected according to the General Data Protection Regulation, GDPR.

## 15. OPERATING AND FINANCIAL MATTERS

The project was initiated by Principal investigator MD Charlotte Lynggaard and Sponsor Professor Christian von Buchwald, the ENT Department of Rigshospitalet. Neither Charlotte Lynggaard nor the others in the research group behind the project have any economic interest in the conduct or outcome of the project. The project's operating expenses will be covered by funds given by private foundations. The funds will be deposited into a fund account (fondskonto) within the Fund Administration (Fondsadministrationen), Rigshospitalet, Copenhagen, Denmark, which is subject to the hospital's audit. The project is not funded by pharmaceutical companies.

### 15.1 Compensation

No financial compensation will be offered to the participants of the study.

It is possible to file a complaint and to receive compensation in accordance to the Danish rules in the health services "Lov om klage- og erstatningsadgang". The participants are covered by the Danish Patient Insurance and through the legislation on compensation after injury associated with pharmaceutical therapy. The latter is described further in the pamphlet "før du beslutter dig".

## 16. CHANGES TO PROTOCOL

The clinical procedures may be changed if the principal investigator and sponsor agree to the changes. If the changes are substantial, both the Ethics Committee and Competent Authorities must approve changes before they can be implemented. All substantial changes must be documented by protocol amendments and rewritten full protocols, if applicable.

## 17. PROPOSED DURATION OF RESEARCH AND DISSEMINATION PLAN

### 17.1 Location and Timing of Study

This study will be conducted at University Hospital of Copenhagen, Rigshospitalet, Denmark. It is expected to start in 4Q 2018 and have a duration of 24 months. Expected duration may be extended. The timetable of the study is as described in appendix II.

## 17.2 Research results

The findings will be disseminated through conference presentations and scientific papers in medical journals. Data will be merged into a series of articles for publication in an international, scientific journal. No data from the study will be published without the involvement and approval by both Charlotte Lynggaard (the lead researcher and PI) and Christian von Buchwald (sponsor). Positive, negative, as well as inconclusive results will be submitted as quickly as possible, in a professional manner and by the General Data Protection Regulation. The project will be recorded prospectively at Clinicaltrials.gov, after the ethical approval. The participants enrolled in the study will be offered to receive information about the results obtained during the study.

All publications will follow the Consolidated Standards of Reporting Trials ( CONSORT) statement[89]. Authorship of any publications resulting from this project will be determined on the basis of the Recommendations for the Conduct, Reporting, Editing, and Publication of Scholarly Work in Medical Journals as stated by the International Committee of the Medical Journal Editors (ICMJE), August 2013, which states that authorship should be based on the following four criteria:

1. Substantial contributions to the conceptions or design of the work; or the acquisition, analysis or interpretation of data of the work
2. Drafting the work or revising it critically for important intellectual content
3. Final approval of the version to be published
4. Agreement to be accountable for all aspects of the work in ensuring the questions related to the accuracy or integrity of any part of the work are appropriately investigated and resolved.

## 18. THE AVAILABILITY OF INFORMATION FOR PARTICIPANTS

The subjects are guaranteed access to more information about the project.
Contact: MD, Ph.D. student Charlotte D. Lynggaard, ENT Department, Rigshospitalet, Copenhagen University Hospital, email: clyn0015@regionh.dk

## 19. DISCONTINUATION OF STUDY

The trial will be discontinued for individual participants in cases where they wish to be withdrawn from the current protocol or in exceptional circumstances where it is impossible to complete the experiment. Likewise, extraordinary events that render the project incomplete in its entirety will lead to withdrawal for all ongoing trials participants. Should data not be accepted for publication in a scientific journal, they will be made public on e.g. the University or Hospital website.

## 20. INSURANCE

The participants are covered in accordance with the regulations of the Danish Patient Insurance Association.

## 21. APPENDICES

# Study flow chart MESRIX-SAFETY

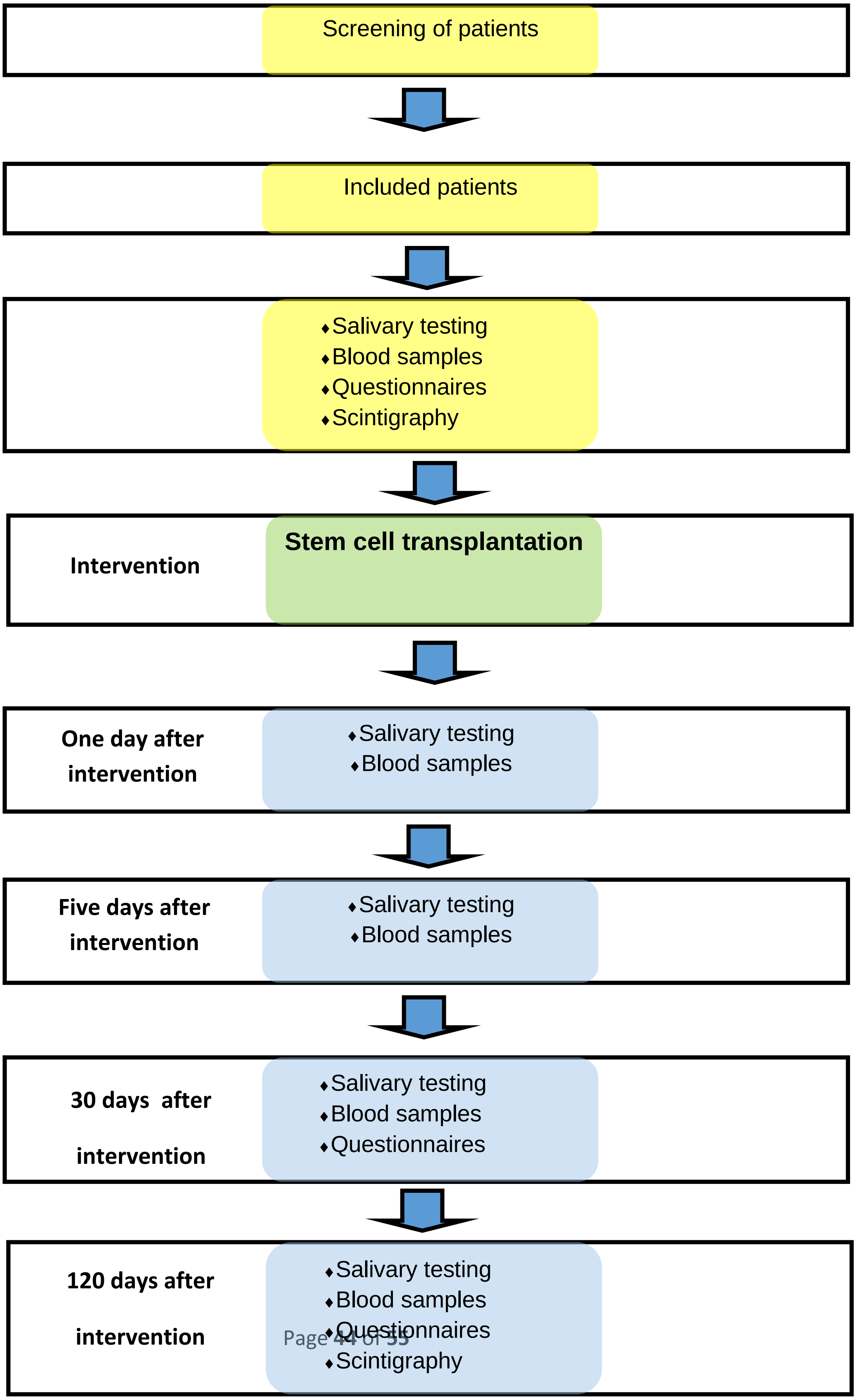

## Appendix II: Table 2: Schedule of enrolment, interventions and assessments

| | STUDY PERIOD | | | | |
|---|---|---|---|---|---|
| | Enrolment | Allocation | Post-allocation | | Close-out |
| VISIT | 1 | 2 | 3 | 4 | 5 |
| TIMEPOINT** | Screening Days -60 to 1 | Intervention Day 1 | Follow-up 1 day after intervention | Follow-up 1 months after intervention | Follow-up 4 months after intervention |
| ENROLMENT: | | | | | |
| Eligibility screen | X | | | | |
| Informed consent | X | | | | |
| Blood sample | X | | | | X |
| (transmissible disease) Allocation | | | | | |
| INTERVENTIONS: | | | | | |
| ASCs | | X | | | |
| | | | | | |
| ASSESSMENTS: | | | | | |
| Sialometry | X | | X | X | X |
| Questionnaires | X | X | X | X | X |
| Blood samples | | X | X | X | X |
| (immune response) | X | | | | X |
| Scintigraphy | X | | X | X | X |
| | | | | | |
| Adverse events Concomitant Medications | X | X | X | X | X |

**Appendix III: Table 3: Schedule of long-term Safety and Efficacy follow-up (S&E-Follow-up)**

| STUDY FOLLOW-UP | | |
|---|---|---|
| VISIT | 1 | 2 |
| TIMEPOINT** | 1 year after Intervention | 2 years after intervention |
| SAFETY<br>ENT examination<br>Blood samples<br>(transmissible disease)<br>Blood samples<br>(immune response)<br>Malignancies formations<br>Emerging diseases | | |
| | X | X |
| | X | |
| | X | X |
| | | |
| | X | X |
| EFFICACY<br>Sialometry<br>QoL Questionnaires | | |
| | X | X |
| | X | X |

| | STUDY PERIOD | | | | |
|---|---|---|---|---|---|
| | Enrolment | Allocation | Post-allocation | | Close-out |
| VISIT | 1 | 2 | 3 | 4 | 5 |
| TIMEPOINT** | Screening<br>Days -60 to 1 | Intervention<br>Day 1 | Follow-up<br>1 day after intervention | Follow-up<br>1 months after intervention | Follow-up<br>4 months after intervention |
| ENROLMENT:<br>Eligibility screen<br>Informed consent<br>Blood sample<br>(transmissible disease)<br>Allocation | | | | | |
| | X | | | | |
| | X | | | | |
| | X | | | | X |
| | | | | | |
| INTERVENTIONS:<br>ASCs | | | | | |
| | | X | | | |
| | | | | | |
| ASSESSMENTS:<br>Sialometry<br>Questionnaires<br>Blood samples<br>(immune response)<br>Scintigraphy<br><br>Adverse events<br>Concomitant Medications | | | | | |
| | X | | X | X | X |
| | X | X | X | X | X |
| | | X | X | X | X |
| | X | | | | X |
| | X | | X | X | X |
| | | | | | |
| | X | X | X | X | X |

**Appendix IV: Table 4: Timetable**

Expected initiation of the study: 01.12.2018

Last visit, last patient (LVLP; 4 months after intervention): 01.12.2020

End of Trial (EOT, last contact to patients and last data collection): 01.12.2026

| Duration | Date | Activity |
|---|---|---|
| 2 months | 01.10.2018-<br>01.12.2018 | Apply for approval of study protocol at DKMA and EC/NVK |
| 6 months | 01.12.2018-<br>01.07.2020 | When permission from DKMA and EC:<br><br>Screening of participants and intervention |
| 18 months | 01.12.2018-<br>01.06.2020 | Interventions<br><br>Initiate follow-up on participants |
| 5 months | 01.07.2020-<br>31.11.2020 | Finish follow-up of participants included in the study<br>End of study period<br>Analysis of data and presentation in scientific articles |

## 22. REFERENCES

1. World Medical Association Declaration of Helsinki. *JAMA*. 2013;310(20):2191. doi:10.1001/jama.2013.281053.

2. Oken MM, Creech RH, Tormey DC, et al. Toxicity and response criteria of the Eastern Cooperative Oncology Group. *Am J Clin Oncol*. 1982;5(6):649-655. http://www.ncbi.nlm.nih.gov/pubmed/7165009. Accessed February 5, 2018.

3. DHMA. *Health Promotion Packages – Introduction and Recommendations*.; 2013.

4. Hussein AA, Helder MN, De Visscher JG, et al. Global incidence of oral and oropharynx cancer in patients younger than 45 years versus older patients: A systematic review ScienceDirect. *Eur J Cancer*. 2017;82:115-127. doi:10.1016/j.ejca.2017.05.026.

5. Carlander A-LF, Grønhøj Larsen C, Jensen DH, et al. Continuing rise in oropharyngeal cancer in a high HPV prevalence area: A Danish population-based study from 2011 to 2014. *Eur J Cancer*. 2017;70:75-82. doi:10.1016/j.ejca.2016.10.015.

6. Jensen AB, Hansen O, Jørgensen K, Bastholt L. Influence of late side-effects upon daily life after radiotherapy for laryngeal and pharyngeal cancer. *Acta Oncol*. 1994;33(5):487-491. http://www.ncbi.nlm.nih.gov/pubmed/7917360. Accessed February 11, 2018.

7. Borras JM, Barton M, Grau C, et al. The impact of cancer incidence and stage on optimal utilization of radiotherapy: Methodology of a population based analysis by the ESTRO-HERO project. *Radiother Oncol*. 2015;116(1):45-50. doi:10.1016/j.radonc.2015.04.021.

8. Jensen SB, Pedersen AML, Vissink A, et al. A systematic review of salivary gland hypofunction and xerostomia induced by cancer therapies: Management strategies and economic impact. *Support Care Cancer*. 2010;18(8):1061-1079. doi:10.1007/s00520-010-0837-6.

9. Jensen DH, Oliveri RS, Trojahn Kølle S-F, et al. Mesenchymal stem cell therapy for salivary gland dysfunction and xerostomia: a systematic review of preclinical studies. *Oral Surg Oral Med Oral Pathol Oral Radiol*. 2014;117(3):335-342.e1. doi:10.1016/j.oooo.2013.11.496.

10. Grønhøj C, Jensen DH, Glovinski P V., et al. First-in-man mesenchymal stem cells for radiation-induced xerostomia (MESRIX): Study protocol for a randomized controlled trial. *Trials*. 2017;18(1):1-10. doi:10.1186/s13063-017-1856-0.

11. Grønhøj C, Jensen DH, Vester-Glowinski P, et al. Safety and Efficacy of Mesenchymal Stem Cells for Radiation-Induced Xerostomia: A Randomized, Placebo-Controlled Phase 1/2 Trial (MESRIX). *Int J Radiat Oncol*. March 2018. doi:10.1016/j.ijrobp.2018.02.034.

12. Pinna R, Campus G, Cumbo E, Mura I, Milia E. Xerostomia induced by radiotherapy: an overview of the physiopathology, clinical evidence, and management of the oral damage. *Ther Clin Risk Manag*. 2015;11:171-188. doi:10.2147/TCRM.S70652.

13. Humphrey SP, Williamson RT. A review of saliva: Normal composition, flow, and function. *J Prosthet Dent*. 2001;85(2):162-169. doi:10.1067/MPR.2001.113778.

14. Avila JL, Grundmann O, Burd R, Limesand KH. Radiation-induced salivary gland dysfunction results from p53-dependent apoptosis. *Int J Radiat Oncol Biol Phys*. 2009;73(2):523-529. doi:10.1016/j.ijrobp.2008.09.036.

15. Ferreira JN, Rungarunlert S, Urkasemsin G, Adine C, Souza GR. Three-Dimensional Bioprinting Nanotechnologies towards Clinical Application of Stem Cells and Their Secretome in Salivary Gland Regeneration. *Stem Cells Int*. 2016. doi:10.1155/2016/7564689.

16. Beier S, Anne J, Pedersen M, Reibel J, Nauntofte B. Xerostomia and hypofunction of the salivary glands in cancer therapy. *Cancer Support Care Cancer*. 2002;11. doi:10.1007/s00520-002-0407-7.

17. Dawes C. Physiological Factors Affecting Salivary Flow Rate, Oral Sugar Clearance, and the Sensation of Dry Mouth in Man. *J Dent Res*. 1987;66:648-653. http://journals.sagepub.com.ep.fjernadgang.kb.dk/doi/pdf/10.1177/00220345870660S107. Accessed January 23, 2018.

18. Vissink A, Jansma J, Spijkervet FKL, Burlage FR, Coppes RP. Oral sequelae of head and neck radiotherapy. *Crit Rev Oral Biol Med*. 2003;14(3):199-212. http://www.ncbi.nlm.nih.gov/pubmed/12799323. Accessed January 23, 2018.

19. Squillaro T, Peluso G, Galderisi U. Clinical Trials with Mesenchymal Stem Cells: An Update. *Cell Transplant*. 2016;25(5):829-848. doi:10.3727/096368915X689622.

20. Friedenstein AJ, Chailakhyan RK, Latsinik N V, Panasyuk AF, Keiliss-Borok I V. Stromal cells responsible for transferring the microenvironment of the hemopoietic tissues. Cloning in vitro and retransplantation in vivo. *Transplantation*. 1974;17(4):331-340. http://www.ncbi.nlm.nih.gov/pubmed/4150881. Accessed January 23, 2018.

21. Caplan A. Mesenchymal stem cells. *J Orthop Res*. 1991;9(5):641-650. doi:10.1002/jor.1100090504.

22. Crisan M, Yap S, Casteilla L, et al. Cell Stem Cell Article A Perivascular Origin for Mesenchymal Stem Cells in Multiple Human Organs. doi:10.1016/j.stem.2008.07.003.

23. Dominici M, Le Blanc K, Mueller I, et al. Minimal criteria for defining multipotent mesenchymal stromal cells. The International Society for Cellular Therapy position statement. 2006. doi:10.1080/14653240600855905.

24. Bourin P, Bunnell BA, Casteilla L, et al. Stromal cells from the adipose tissue-derived stromal vascular fraction and culture expanded adipose tissue-derived stromal/ stem cells: a joint statement of the International Federation for Adipose Therapeutics (IFATS) and Science and the International Society for Cellular Therapy (ISCT). *Cytotherapy*. 2013;15(6):641-648. doi:10.1016/j.jcyt.2013.02.006.

25. Galipeau J, Krampera M, Barrett J, et al. International Society for Cellular Therapy perspective on immune functional assays for mesenchymal stromal cells as potency release criterion for advanced phase clinical trials. doi:10.1016/j.jcyt.2015.11.008.

26. Kastrup J, Schou M, Gustafsson I, et al. Rationale and Design of the First Double-Blind, Placebo-Controlled Trial with Allogeneic Adipose Tissue-Derived Stromal Cell Therapy in Patients with Ischemic Heart Failure: A Phase II Danish Multicentre Study. *Stem Cells Int*. 2017;2017. doi:10.1155/2017/8506370.

27. Gyöngyösi M, Wojakowski W, Navarese EP, À Moye L. Controversies in Cardiovascular Research: Controversies in meta-analyses results on cardiac cell-based regenerative studies. doi:10.1161/CIRCRESAHA.115.307347.

28. Juhl M, Tratwal J, Follin B, et al. Comparison of clinical grade human platelet lysates for cultivation of mesenchymal stromal cells from bone marrow and adipose tissue. *Scand J Clin Lab Invest*. 2016;76(2):93-104. doi:10.3109/00365513.2015.1099723.

29. Baer PC. Adipose-Derived Stem Cells and Their Potential to Differentiate into the Epithelial Lineage. doi:10.1089/scd.2011.0086.

30. Ankrum JA, Ong JF, Karp JM. Mesenchymal stem cells: Immune evasive, not immune privileged. *Nat Biotechnol*. 2014;32(3):252-260. doi:10.1038/nbt.2816.

31. Prockop DJ. The exciting prospects of new therapies with mesenchymal stromal cells. *Cytotherapy*. 2017;19(1):1-8. doi:10.1016/j.jcyt.2016.09.008.

32. Singer NG, Caplan AI. Mesenchymal Stem Cells: Mechanisms of Inflammation. *Annu Rev Pathol Mech Dis*. 2011;6:457-478. doi:10.1146/annurev-pathol-011110-130230.

33. Lombaert I, Movahednia MM, Adine C, Ferreira JN. Concise Review: Salivary Gland Regeneration: Therapeutic Approaches from Stem Cells to Tissue Organoids. *Stem Cells*. 2017. doi:10.1002/stem.2455.

34. Choi JS, An HY, Shin HS, Kim YM, Lim JY. Enhanced tissue remodelling efficacy of adipose-derived mesenchymal stem cells using injectable matrices in radiation-damaged salivary gland model. *J Tissue Eng Regen Med*. 2017. doi:10.1002/term.2352.

35. Lim J-Y, Ra JC, Shin IS, et al. Systemic Transplantation of Human Adipose Tissue-Derived Mesenchymal Stem Cells for the Regeneration of Irradiation-Induced Salivary Gland Damage. Deutsch E, ed. *PLoS One*. 2013;8(8):e71167. doi:10.1371/journal.pone.0071167.

36. Comella K, Bell W. First-in-man intraglandular implantation of stromal vascular fraction and adipose-derived stem cells plus platelet-rich plasma in irradiation-induced gland damage: A case study. *Int Med Case Rep J*. 2017;10:295-299. doi:10.2147/IMCRJ.S142514.

37. Coipeau P, Rosset P, Langonn A, et al. Impaired differentiation potential of human trabecular bone mesenchymal stromal cells from elderly patients. *Cytotherapy*. 2009;11(5):584-594. doi:10.1080/14653240903079385.

38. Neef K, Choi Y-H, Weichel A, et al. The influence of cardiovascular risk factors on bone marrow mesenchymal stromal cell fitness. *Cytotherapy*. 2012;14(6):670-678. doi:10.3109/14653249.2012.663483.

39. Ding D-C, Chang Y-H, Liu H-W, Chu T-Y, Wen Y-T, Tsai R-K. Cisplatin-Impaired Adipogenic Differentiation of Adipose Mesenchymal Stem Cells 1. *Cell Transplant*. 2017;26:1077-1087. doi:10.3727/096368917X694886.

40. Krawiec JT, Weinbaum JS, St. Croix CM, et al. A Cautionary Tale for Autologous Vascular Tissue Engineering: Impact of Human Demographics on the Ability of Adipose-Derived Mesenchymal Stem Cells to Recruit and Differentiate into Smooth Muscle Cells. *Tissue Eng Part A*. 2015;21(3-4):426-437. doi:10.1089/ten.tea.2014.0208.

41. Sher DJ, Adelstein DJ, Bajaj GK, et al. Radiation therapy for oropharyngeal squamous cell carcinoma: Executive summary of an ASTRO Evidence-Based Clinical Practice Guideline. *Pract Radiat Oncol*. 2017;7(4):246-253. doi:10.1016/j.prro.2017.02.002.

42. Le Blanc K, Frassoni F, Ball L, et al. Mesenchymal stem cells for treatment of steroid-resistant, severe, acute graft-versus-host disease: a phase II study. *Lancet (London, England)*. 2008;371(9624):1579-1586. doi:10.1016/S0140-6736(08)60690-X.

43. Hare JM, Difede DL, Rieger AC, et al. Randomized Comparison of Allogeneic Versus Autologous Mesenchymal Stem Cells for Nonischemic Dilated Cardiomyopathy POSEIDON-DCM Trial. https://www-clinicalkey-com.ep.fjernadgang.kb.dk/service/content/pdf/watermarked/1-s2.0-S0735109716369066.pdf?locale=en_US. Accessed January 29, 2018.

44. Panés J, García-Olmo D, Van Assche G, et al. Expanded allogeneic adipose-derived mesenchymal stem cells (Cx601) for complex perianal fistulas in Crohn’s disease: a phase 3 randomised, double-blind controlled trial. *Lancet*. 2016;388(10051):1281-1290. doi:10.1016/S0140-6736(16)31203-X.

45. De La Portilla F, Alba F, García-Olmo D, Herrerías JM, González FX, Galindo A. Expanded allogeneic adipose-derived stem cells (eASCs) for the treatment of complex perianal fistula in Crohn’s disease: results from a multicenter phase I/IIa clinical trial. 2012. doi:10.1007/s00384-012-1581-9.

46. Xu J, Wang D, Liu D, et al. Allogeneic mesenchymal stem cell treatment alleviates experimental and clinical Sjögren syndrome. *Blood*. 2012;120(15):3142-3151. doi:10.1182/blood-2011-11-391144.

47. Wang D, Zhang H, Liang J, et al. A Long-Term Follow-Up Study of Allogeneic Mesenchymal Stem/Stromal Cell Transplantation in Patients with Drug-Resistant Systemic Lupus Erythematosus. *Stem Cell Reports*. 2018;10(3):933-941. doi:10.1016/j.stemcr.2018.01.029.

48. Ramireddy A, Brodt CR, Mendizabal AM, et al. Effects of Transendocardial Stem Cell Injection on Ventricular Proarrhythmia in Patients with Ischemic Cardiomyopathy: Results from the POSEIDON and TAC-HFT Trials. *Stem Cells Transl Med*. 2017;6(5):1366-1372. doi:10.1002/sctm.16-0328.

49. Chullikana A, Majumdar A Sen, Gottipamula S, et al. Randomized, double-blind, phase I/II study of intravenous allogeneic mesenchymal stromal cells in acute myocardial infarction. *Cytotherapy*. 2015;17(3):250-261. doi:10.1016/j.jcyt.2014.10.009.

50. Kastrup J, Haack-Sørensen M, Juhl M, et al. Cryopreserved Off-the-Shelf Allogeneic Adipose-Derived Stromal Cells for Therapy in Patients with Ischemic Heart Disease and Heart Failure—A Safety Study. *Stem Cells Transl Med*. 2017;6(11):1963-1971. doi:10.1002/sctm.17-0040.

51. Molendijk I, Bonsing BA, Roelofs H, et al. Allogeneic Bone MarrowLDerived Mesenchymal Stromal Cells Promote Healing of Refractory Perianal Fistulas in Patients With Crohn's Disease. doi:10.1053/j.gastro.2015.06.014.

52. Panés J, García-Olmo D, Assche G Van, et al. Long-term Efficacy and Safety of Stem Cell Therapy (Cx601) for Complex Perianal Fistulas in Patients With Crohn's Disease. https://www.clinicalkey.com/service/content/pdf/watermarked/1-s2.0-S0016508517367264.pdf?locale=en_US. Accessed May 21, 2018.

53. Hare JM, Traverse JH, Henry TD, et al. A Randomized, Double-Blind, Placebo-Controlled, Dose- Escalation Study of Intravenous Adult Human Mesenchymal Stem Cells (Prochymal) After Acute Myocardial Infarction. doi:10.1016/j.jacc.2009.06.055.

54. Xu J, Wang D, Liu D, et al. Allogeneic mesenchymal stem cell treatment alleviates experimental and clinical Sjögren syndrome. *Blood*. 2012;120(15):3142-3151. doi:10.1182/blood-2011-11-391144.

55. Wang D, Akiyama K, Zhang H, et al. Double allogenic mesenchymal stem cells transplantations could not enhance therapeutic effect compared with single transplantation in systemic lupus erythematosus. *Clin Dev Immunol*. 2012;2012:273291. doi:10.1155/2012/273291.

56. Hass R, Kasper C, Böhm S, Jacobs R. Different populations and sources of human mesenchymal stem cells (MSC): A comparison of adult and neonatal tissue-derived MSC. *Cell Commun Signal*. 2011;9(1):12. doi:10.1186/1478-811X-9-12.

57. Berglund AK, Fortier LA, Antczak DF, Schnabel L V. Immunoprivileged no more: Measuring the immunogenicity of allogeneic adult mesenchymal stem cells. *Stem Cell Res Ther*. 2017;8(1):1-7. doi:10.1186/s13287-017-0742-8.

58. García-Sancho J, Sánchez A, Vega A, Noriega DC, Nocito M. Influence of HLA Matching on the Efficacy of Allogeneic Mesenchymal Stromal Cell Therapies for Osteoarthritis and Degenerative Disc Disease. doi:10.1097/TXD.0000000000000724.

59. Von Bahr L, Batsis I, Moll G, et al. Analysis of tissues following mesenchymal stromal cell therapy in humans indicates limited long-term engraftment and no ectopic tissue formation. *Stem Cells*. 2012;30(7):1575-1578. doi:10.1002/stem.1118.

60. Wang Z-H, Yan C, Zhang Z-Y, et al. Radiation-induced volume changes in parotid and submandibular glands in patients with head and neck cancer receiving postoperative radiotherapy: A longitudinal study. *Laryngoscope*. 2009. doi:10.1002/lary.20601.

61. Cheng SCH, Wu VWC, Kwong DLW, Ying M. Assessment of post-radiotherapy salivary glands. *Br J Radiol*. 2011;84(1001):393-402. doi:10.1259/bjr/66754762.

62. Jongerius PH, Joosten F, Hoogen FJA, Gabreels FJM, Rotteveel JJ. The Treatment of Drooling by Ultrasound- Guided Intraglandular Injections of Botulinum Toxin Type A Into the Salivary Glands. *Laryngoscope*. 2003;113(January):107-111.

63. Eisbruch A, Rhodus N, Rosenthal D, et al. How should we measure and report radiotherapy-induced xerostomia? *Semin Radiat Oncol*. 2003;13(3):226-234. doi:10.1016/S1053-4296(03)00033-X.

64. Navazesh M, Christensen CM. A Comparison of Whole Mouth Resting and Stimulated Salivary Measurement Procedures. http://journals.sagepub.com.ep.fjernadgang.kb.dk/doi/pdf/10.1177/00220345820610100901. Accessed February 5, 2018.

65. Anjos DA, Etchebehere ECSC, Santos AO, et al. Normal values of [99mTc]pertechnetate uptake and excretion fraction by major salivary glands. *Nucl Med Commun*. 2006;27(4):395-403. doi:10.1097/01.mnm.0000202864.52046.b1.

66. Kohn WG, Ship JA, Atkinson JC, Patton LL, Fox PC. Salivary gland 99mTc-scintigraphy: a grading scale and correlation with major salivary gland flow rates. *J Oral Pathol Med*. 1992;21(2):70-74. http://www.ncbi.nlm.nih.gov/pubmed/1313502. Accessed February 9, 2018.

67. Bardow A, Madsen J NB. The bicarbonste concentration in the human saliva does not exceed the plasma level under normal conditions. https://search-proquest-com.ep.fjernadgang.kb.dk/docview/229535431/fulltextPDF/3BF276C62AF24CD0PQ/1?accountid=13607. Accessed February 5, 2018.

68. Lajer C, Buchwald C, Nauntofte B, Specht L, Bardow A, Jensdottir T. Erosive potential of saliva stimulating tablets with and without fluoride in irradiated head and neck cancer patients. *Radiother Oncol*. 2009;93:534-538. doi:10.1016/j.radonc.2009.06.028.

69. Bruvo M, Moe D, Kirkeby S, Vorum H, Bardow A. Individual Variations in Protective Effects of Experimentally Formed Salivary Pellicles. *Caries Res*. 2009;43:163-170. doi:10.1159/000213887.

70. LEXNER MO, BARDOW A, HERTZ JM, ALMER L, NAUNTOFTE B, KREIBORG S. Whole saliva in X-linked hypohidrotic ectodermal dysplasia. *Int J Paediatr Dent*. 2007;17(3):155-162. doi:10.1111/j.1365-263X.2006.00812.x.

71. Prasad G, McCullough M. Chemokines and cytokines as salivary biomarkers for the early diagnosis of oral cancer. *Int J Dent*. 2013;2013:813756. doi:10.1155/2013/813756.

72. Jensen K, Jensen AB, Grau C. A cross sectional quality of life study of 116 recurrence free head and neck cancer patients. The first use of EORTC H&N35 in Danish. *Acta Oncol (Madr)*. 2006;45(1):28-37. doi:10.1080/02841860500417536.

73. Grønhøj Larsen, Christian, Jensen, David, Mafioso, Roberto, Fischer-Nielsen, Anne, Specht, Lena, Fog, Lea Munthe, Kiss, Katalin, Jensen, Siri Beier, Jensen, Allan Bardow and Buchwald C von. Safety and Efficacy of Autologous Tissue-derived Mesenchymal Stem Cells for

Radiation- Induced Xerostomia: A Randomized, Placebo-Controlled Phase I/II Trial (MESRIX). *Int J Radiat Oncol Biol Phys*. 2018;(Accepted):1-17.

74. Qiao L, Xu Z-L, Zhao T-J, Ye L-H, Zhang X-D. Dkk-1 secreted by mesenchymal stem cells inhibits growth of breast cancer cells via depression of Wnt signalling. doi:10.1016/j.canlet.2008.04.032.

75. Cousin B, Ravet E, Poglio S, et al. Adult Stromal Cells Derived from Human Adipose Tissue Provoke Pancreatic Cancer Cell Death both In Vitro and In Vivo. *Vitr Vivo PLoS ONE*. 2009;4(7). doi:10.1371/journal.pone.0006278.

76. Ramasamy R, Lam E-F, Soeiro I, Tisato V, Bonnet D, Dazzi F. Mesenchymal stem cells inhibit proliferation and apoptosis of tumor cells: impact on in vivo tumor growth. *Leukemia*. 2007;21:304-310. doi:10.1038/sj.leu.2404489.

77. Bian Z-Y, Fan Q-M, Li G, Xu W-T, Tang T-T. Human mesenchymal stem cells promote growth of osteosarcoma: Involvement of interleukin-6 in the interaction between human mesenchymal stem cells and Saos-2. *Cancer Sci*. 2010;101(12):2554-2560. doi:10.1111/j.1349-7006.2010.01731.x.

78. Kucerova L, Matuskova M, Hlubinova K, Altanerova V, Altaner C. Tumor cell behaviour modulation by mesenchymal stromal cells. *Mol Cancer*. 2010;9. http://www.molecular-cancer.com/content/9/1/129. Accessed February 5, 2018.

79. Prockop DJ, Brenner M, Fibbe WE, et al. Defining the risks of mesenchymal stromal cell therapy. *Cytotherapy*. 2010;12(5):576-578. doi:10.3109/14653249.2010.507330.

80. Casiraghi F, Remuzzi G, Abbate M, Perico N. Multipotent Mesenchymal Stromal Cell Therapy and Risk of Malignancies. *Stem Cell Rev Reports*. 2013;9(1):65-79. doi:10.1007/s12015-011-9345-4.

81. Toyserkani NM, Jørgensen MG, Tabatabaeifar S, Jensen CH, Sheikh SP, Sørensen JA. Concise Review: A Safety Assessment of Adipose-Derived Cell Therapy in Clinical Trials: A Systematic Review of Reported Adverse Events. *Stem Cells Transl Med*. 2017;6(9):1786-1794. doi:10.1002/sctm.17-0031.

82. Qayyum AA, Haack-Sørensen M, Mathiasen AB, Jørgensen E, Ekblond A, Kastrup J. Adipose-derived mesenchymal stromal cells for chronic myocardial ischemia (MyStromalCell Trial): study design. *Regen Med*. 2012;7(3):421-428. doi:10.2217/rme.12.17.

83. M.D Herreros, M. Garcia-Arranz, H. Guadakajara, P.De-La-Quintana DG-O and the FCG. Autologous Expanded Adipose-Derived Stem Cells for the Treatment of Compelx Crytoglandular Perianal Fistulas: A Phase III Randomized Clinical Trial( FATT 1: Fistula Advanced Therapy Trial 1) and Long-term Evaluation. http://ovidsp.uk.ovid.com.ep.fjernadgang.kb.dk/sp-3.27.2b/ovidweb.cgi?WebLinkFrameset=1&S=HFHJPDHKEAHFHIGLFNFKBCOFOALJAA00&returnUrl=ovidweb.cgi%3F%26Full%2BText%3DL%257cS.sh.27.28%257c0%257c00003453-201207000-00005%26S%3DHFHJPDHKEAHFHIGLFNFKBCOFOALJAA00&. Accessed February 5, 2018.

84. Trojahn Kølle SF, Oliveri RS, Glovinski P V., et al. Pooled human platelet lysate versus fetal bovine serum-investigating the proliferation rate, chromosome stability and angiogenic potential of human adipose tissue-derived stem cells intended for clinical use. *Cytotherapy*. 2013;15(9):1086-1097. doi:10.1016/j.jcyt.2013.01.217.

85. Kølle S-FT, Fischer-Nielsen A, Mathiasen AB, et al. Enrichment of autologous fat grafts with ex-vivo expanded adipose tissue-derived stem cells for graft survival: a randomised placebo-controlled trial. *Lancet*. 2013;382(9898):1113-1120. doi:10.1016/S0140-6736(13)61410-5.

86. Simmons RK, Baum BJ. Transferring Genes to Salivary Glands. *J Dent Educ*. 2001. http://www.jdentaled.org/content/65/9/907.full.pdf. Accessed February 5, 2018.

87. Wey Yong K, Kamarul Zaman Wan Safwani W, Xu F, Abu Bakar Wan Abas W, Ru Choi J, Pingguan-Murphy B. Cryopreservation of Human Mesenchymal Stem Cells for Clinical Applications: Current Methods and Challenges. doi:10.1089/bio.2014.0104.

88. Villatoro AJ, Fernández V, Claros S, Rico-Llanos GA, Becerra J, Andrades JA. Use of adipose-derived mesenchymal stem cells in keratoconjunctivitis sicca in a canine model. *Biomed Res Int*. 2015;2015. doi:10.1155/2015/527926.

89. Boutron I, Altman DG, Moher D, Schulz KF, Ravaud P. CONSORT Statement for Randomized Trials of Nonpharmacologic Treatments: A 2017 Update and a CONSORT Extension for Nonpharmacologic Trial Abstracts. *Ann Intern Med*. 2017;167(1):40. doi:10.7326/M17-0046.
